# Protein-based layer-by-layer films for biomedical applications

**DOI:** 10.1039/d3sc06549a

**Published:** 2024-05-14

**Authors:** Muhammad Haseeb Iqbal, Halima Kerdjoudj, Fouzia Boulmedais

**Affiliations:** a Université de Strasbourg, CNRS, Institut Charles Sadron UPR 22, Strasbourg Cedex 2 67034 France haseebntu1@gmail.com fouzia.boulmedais@ics-cnrs.unistra.fr; b Université de Reims Champagne Ardenne 51100 Reims France

## Abstract

The surface engineering of biomaterials is crucial for their successful (bio)integration by the body, *i.e.* the colonization by the tissue-specific cell, and the prevention of fibrosis and/or bacterial colonization. Performed at room temperature in an aqueous medium, the layer-by-layer (LbL) coating method is based on the alternating deposition of macromolecules. Versatile and simple, this method allows the functionalization of surfaces with proteins, which play a crucial role in several biological mechanisms. Possessing intrinsic properties (cell adhesion, antibacterial, degradable, *etc.*), protein-based LbL films represent a powerful tool to control bacterial and mammalian cell fate. In this article, after a general introduction to the LbL technique, we will focus on protein-based LbL films addressing different biomedical issues/domains, such as bacterial infection, blood contacting surfaces, mammalian cell adhesion, drug and gene delivery, and bone and neural tissue engineering. We do not consider biosensing applications or electrochemical aspects using specific proteins such as enzymes.

## Abstract 2

Based on alternating deposition of macromolecules, the layer-by-layer (LbL) method allows the functionalization of surfaces. Possessing intrinsic properties, protein-based LbL films are a powerful tool to control bacterial and mammalian cell fate.

## Graphical abstract

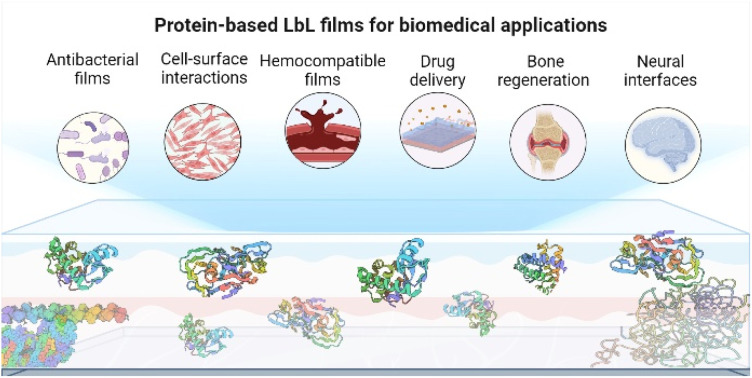

## Introduction

Biomaterials, such as implantable medical devices, are widely used in reconstructive and regenerative medicine. They are intended to be in contact with body fluids and/or tissues. Engineering the surface of such biomaterials is of vital importance for their successful (bio)integration by the body, *i.e.*, colonization by the specific cell of the replaced tissue and/or preventing fibrosis and bacterial colonization. Among the surface functionalization methods, the layer-by-layer (LbL) method is simple to implement at room temperature using aqueous solutions and is potentially applicable in industry. Versatile, this method is based on the alternated deposition of oppositely charged polyelectrolytes allowing the development of biocompatible and bioactive nano or submicron films. Since 1992 with the first paper of Gero Decher using polyelectrolytes,^1^ a tremendous interest has been shown with thousands of annual publications with a wide spectrum of applications in healthcare. A variety of (macro)molecules or nano/micromaterials (synthetic or natural polymers, nanostructures, proteins, or enzymes) have been used to develop LbL nanofilms over time. Several reviews report on the physical chemistry and applications of synthetic and natural polymer-based LbL films.^2,3^ Most of these works focus on the physical-chemistry aspects of LbL films and their use in specific biomedical applications.

Proteins play a crucial role in several biological mechanisms, such as DNA replication, metabolic reactions, molecule transport, cellular adhesion, *etc.* The use of proteins in LbL assembly is limited, despite the growing potential, due to their structural complexities. Besides electrostatic interactions, hydrogen bonding and hydrophobic interactions play a crucial role in protein-based LbL. Frequent literature is available on enzyme-based films for biosensing applications^4,5^ and only minor or scattered patches of highlights exist on non-enzyme proteins in general reviews. Regarding the physical chemistry of protein-based films, we recommend the review from Dupon-Gillain and co-workers, which gives a guide on the choice of polyelectrolytes and the building conditions that lead to their successful growth.^2^ LbL films based solely on proteins are rare due to specific interactions limiting the possible combinations, such as fibronectin with gelatin (Gel) or elastin (ELP),^6^ or to their ampholyte nature limiting the construction pH as for gelatin.^7^ It can be noticed that type I collagen (COL)/fibronectin (Fn) LbL films were reported to show unsuccessful build-up despite using three different buffers and incorporation of an anchoring layer.^8^ COL,^9–14^ Gel,^6,7,15–20^ Fn,^6,21,22^ fibrinogen,^21^ ELP,^6,23^ laminin,^24^ lysozyme (Ly),^25,26^ casein,^26^ and bovine albumin (BSA)^24,27,28^ were mostly associated with synthetic polymers, polysaccharides, or tannic acid (TA, polyphenol).

In this review after a general introduction to the LbL technique, we will mainly focus on the LbL films developed using proteins, as at least one component, and addressing different biomedical applications: as antibacterial coatings, to promote mammalian cell adhesion, blood contacting surfaces, drug and gene delivery, bone tissue engineering, and neural tissue engineering. We are not considering the biosensing application or electrochemistry aspects using specific proteins such as enzymes.

## Principle of layer-by-layer films

**Fig. 1:**
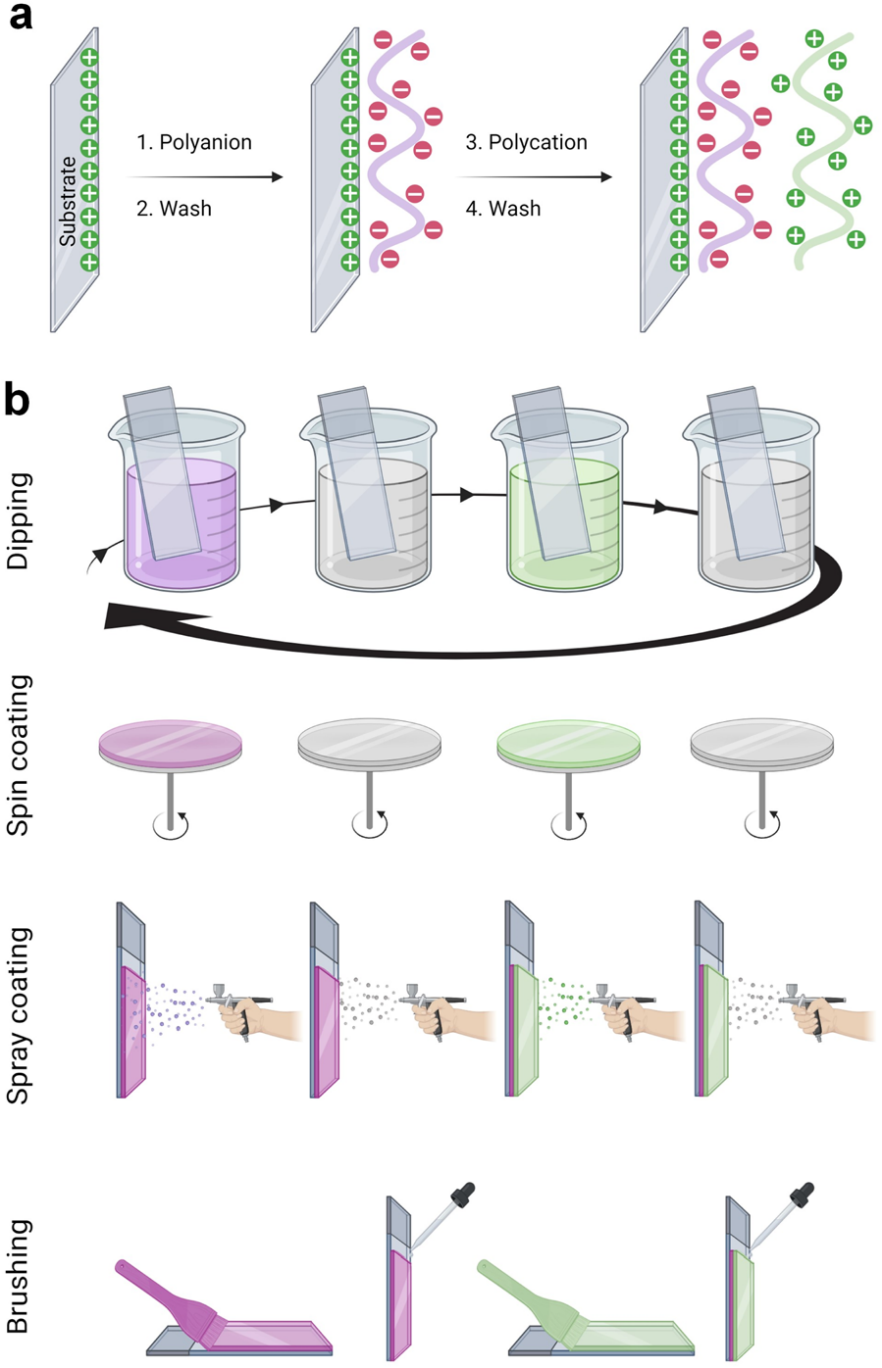
Layer-by-layer (LbL) method and different processes of build-up. (a) Schematic representation of the alternated deposition of polyanions and polycations, each deposition is followed by a rinsing step. (b) The build-up of LbL films can be achieved by various processes like dipping, spin coating, spray coating, and brushing.

The LbL assembly was introduced in 1992 by Gero Decher using electrostatic interactions between oppositely charged polyelectrolytes, polyanion, and polycation, to obtain polyelectrolyte multilayer films (Fig. 1a).^1,29^ Poly(styrene sulfonate)/poly(allylamine hydrochloride) (PSS/PAH) LbL films were deposited by the dipping method. After a silanization step to obtain a positively charged surface, the substrate was dipped in the polyanion (PSS) aqueous solution. After the rinsing step to remove weakly bound molecules, the surface became negatively charged by overcompensation of charges. With the adsorption of the polycation (PAH) layer, a positively charged surface was obtained by reversing the charge. This process can be repeated to obtain the desired film thickness. Later, the process was done on raw substrate, usually negatively charged. PSS/PAH LbL films were described to favour hepatocyte adhesion^30^ and progenitor endothelial cell maturation.^31^

### LbL films: linear *vs.* exponential growth

The growth of PSS/PAH films is linear, *i.e.*, the thickness and the mass increment of the adsorbed pair of layers is constant whatever the number of adsorption cycles. The adsorbed polyelectrolytes interact only with the top outer layer of the film forming stratified films.^32^ Different parameters such as the pH, temperature, ionic strength, type of salt, and the characteristics of each polyelectrolyte strongly influence the build-up of the films. Thus, depending on the conditions of the build-up, the polyelectrolytes can adopt different conformations influencing the growth of the LbL films, *i.e.*, the mass adsorbed and their thickness. At low ionic strength, they present a “flat” or rigid rod-like conformation since the polyelectrolyte charges repel each other resulting in a thin deposited thickness. At high ionic strength, polyelectrolytes adopt a “loopy” conformation by charge screening resulting in a large deposited thickness. Strong polyelectrolytes are fully charged regardless of the pH, but the weak polyelectrolytes, mostly containing amine or carboxylic groups, are pH sensitive possessing a p*K*_a_. Below the p*K*_a_, weak polyanions are protonated with low density of charges and a flat conformation. Above the p*K*_a_, the density of charges per chain increases leading to a loopy conformation.

Other types of LbL build-up were reported later. The super-linear growth (faster growth than linear) of LbL films was attributed to an increase of the film roughness along the whole build-up process.^33,34^ Exponentially growing films were first observed by Hubbel and co-workers.^35^ The film thickness and mass increase exponentially with the number of adsorption cycles. Picart and co-workers explained the growth by the diffusion of at least one of the polyelectrolytes into the entire film during each deposition step leading to a reservoir of polymer chains able to diffuse out and complex with the oppositely charged polymer chains at the next deposition step. These complexes form an additional layer on the top of the film, thus the mass (and thickness) of this layer is proportional to the film thickness (the size of the reservoir containing the polyelectrolyte chains): signature of an exponential build-up.^36–38^ After several deposition steps, exponentially growing films enter a linear growth phase.^39,40^ This could be due to a restructuring of the film that gradually prohibits the diffusion of polyelectrolytes throughout the entire film and limits it to a constant film thickness leading to linear growth. The growth regime of LbL films is related to the interaction strength between the two polyelectrolytes. The build-up process of linearly growing films is based on polyelectrolyte complexation both enthalpically and entropically driven. On the other hand, the complexation is entirely entropically driven in the case of exponentially growing films.^41^ Protein-based LbL films usually have linear growth and a few have exponential growth, especially when associated with a polysaccharide.^2^

### Other interactions

The hydrophobic interactions play a significant role in the adsorption of proteins favouring their LbL build-up.^42,43^ LbL films based on other interactions were also reported based on hydrogen bonds,^44^ covalent bonds,^45,46^ and bio-specific interactions.^47–49^ Apart from electrostatic interactions, hydrogen bonding is one of the most studied driving forces because it allows the insertion of uncharged molecules that can operate as hydrogen bonding donors and acceptors.^50^ Built at a specific pH, where polyelectrolytes are not charged, H-bonded LbL films are highly sensitive to post-build-up treatment, such as pH and temperature but not the ionic strength. For example, poly(acrylic acid)/poly(4-vinyl pyridine) LbL films disassemble above pH 6.9 due to ionization of carboxylic groups of poly(acrylic acid).^51^ Thanks to the pH responsiveness, H-bonded LbL films or capsules with controlled degradation are extensively used for drug delivery systems.^52^ Thanks to specific lectin–carbohydrate interaction, LbL microcapsules were fabricated from Concanavalin A, a plant lectin, and glycogen, a polysaccharide.^49^ Protein LbL films composed of avidin and biotin-labelled antibody were also prepared using the high specific binding constant (*K*_a_: ≈10^15^ M^−1^) between avidin and biotin.

The film stability and rigidity can be enhanced by covalent bonds at each step^53^ or after the build-up.^9,54–56^ Haemoglobin-based microcapsules were obtained by alternated deposition with a cross-linker, glutaraldehyde. The build-up was based on the Schiff base reaction between the amino sites of haemoglobin and aldehyde groups of the cross-linker.^53^ To avoid the dissolution, the cross-linking of the collagen-based films was performed using carbodiimide,^9^ glutaraldehyde,^54^ or genipin.^55,56^ More chemically and mechanically stable, covalent cross-linked films are probably the most used films in various applications like cell-surface interactions, drug delivery, surface patterning, electrooptical devices, catalytic substrates, anti-friction *etc.*^57^

### Deposition processes

LbL films have been developed using various methods namely, dipping,^1^ spin-coating,^58^ spray-coating,^59^ and more recently the brushing^60^ method (Fig. 1b). The dip coating method can be used on various substrates of practically any possible shape, with economical consumption of products *i.e.*, the same solutions can be used for several deposition steps. It offers a wide selection of materials, from synthetic to biological molecules, which can be deposited, and fine control on the film structure, thickness, and functionality. However, each deposition step requires prolonged time, generally between 5 to 20 min for each deposition or rinsing.

The problem with long deposition time was addressed by using spin-coating to develop LbL films on planar surfaces. This method involves dispensing a droplet of polyelectrolyte solution in the centre of the surface and rotating the surface at a controlled speed. The rotating surface exerts a centrifugal force on the polyelectrolyte solution causing it to spread radially outwards on the surface. Under this motion, the excess material is ejected off the surface, leaving behind a uniformly deposited layer. The required time to deposit one layer is typically around 1 min which is extremely fast compared to the dip-coating. Generally, there is no rinsing step required in this method which allows a faster film deposition. The rotation speed, viscosity or concentration, and polyelectrolyte type impact the film's roughness and thickness. A high concentration of polyelectrolytes and low rotation speed led to thicker LbL films.^61^ Though, spin-coating allows fast deposition time but is limited to a planar surface with reasonable size.

The spraying method was first reported by Schlenoff *et al.* to develop PSS and poly(diallyl dimethyl ammonium chloride) LbL films.^59^ Polyelectrolyte solutions and rinsing water were sprayed on the substrate held vertically to allow solution drainage under gravity. Like spin-coating, this is also a fast method that allows the coating of larger surface areas, but this process consumes a high amount of solution.

Recently, an attractive and simple method to develop LbL films has been proposed by brushing. Unlike the dip-coating, this method is tremendously fast requiring only a few seconds of deposition time for one layer, and does not require specific and expensive material. The brushing process allowed to design of chitosan/alginate multilayer films for drug delivery,^62^ and collagen/tannic acid for myoblast differentiation into myotubes.^63^ The solution concentrations and the brush type were reported to impact the film thickness and nanotopography.

**Scheme 1:**
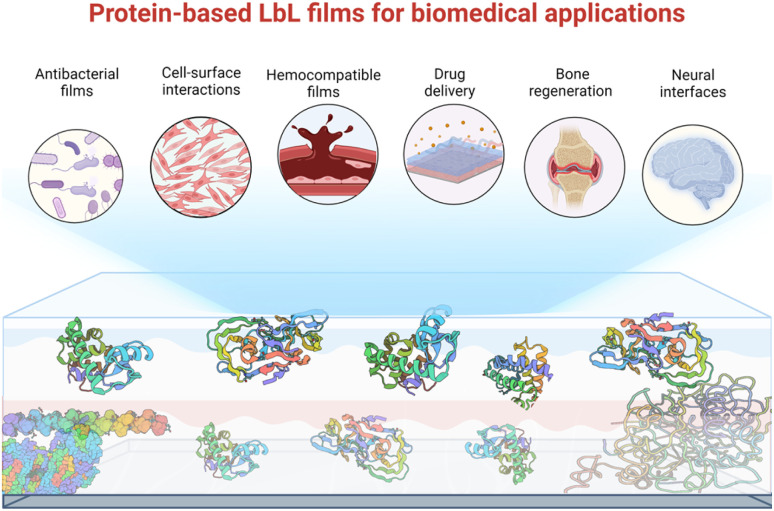
Schematic representation of protein-based LbL films and their various biomedical application addressed in this perspective.

In 2001, the first protein-based LbL film was reported using COL, a fibrillar protein mostly found in extracellular matrix, to promote cell adhesion on biomaterial surfaces.^64^ Since 2006, the LbL assembly of extracellular matrix proteins gained huge interest in developing multifunctional biomaterials despite various difficulties associated with their use.^65^ In the following, we will present the major biomedical issues and how the LbL films addressed them (Scheme 1).

## Antibacterial LbL films

**Scheme 2:**
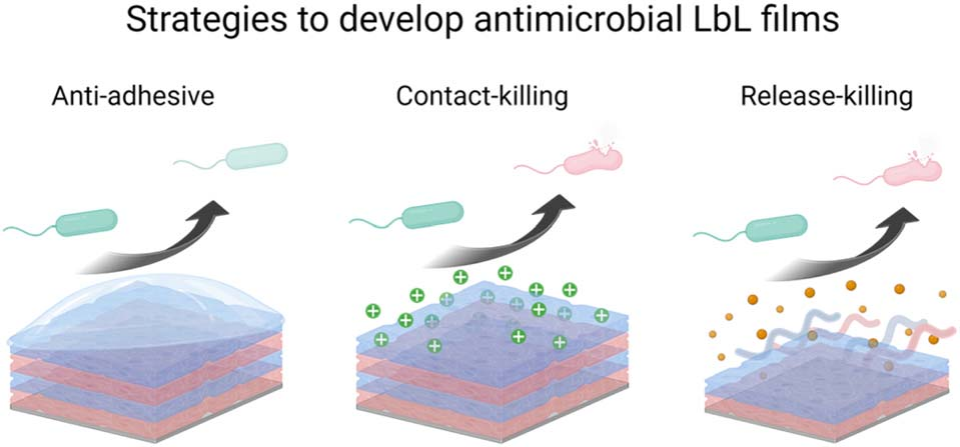
Schematic representation of the three strategies of antibacterial LbL films.

Microbial contamination and infection pose severe health and economic threats, mainly because of the increasing resistance of bacteria toward conventional antibiotic treatments. The antibacterial surface design has attracted great attention over the past few decades. In this context, LbL films have been widely developed to achieve mainly three properties: (i) antiadhesive films to prevent bacteria attachment, (ii) contact-killing films to inactivate bacteria upon contact, and (iii) release-killing LbL films to leach out antimicrobial agents (Scheme 2). A comprehensive description of these coatings using organic and inorganic molecules, polyelectrolytes, antimicrobial peptides, nanoparticles, *etc.* is reviewed elsewhere.^66^

### Adhesion-resistant films

To prevent the early attachment of bacteria and further biofilm formation, adhesion-resistant LbL films are usually highly hydrophilic films^67,68^ or possess specific stiffnesses to prevent bacterial adhesion.^69,70^ To enhance their hydrophilicity, synthetic polyelectrolytes have to be modified by poly(ethylene glycol)^67,68^ or phosphoryl choline^71^ before deposition to build adhesion-resistant LbL. In contrast, hyaluronic acid (HA)-based LbL films present an intrinsic high hydrophilicity preventing the adhesion of bacteria. Both synthetic and natural polyelectrolytes-based LbL present specific film rigidity able to present bacterial adhesion.^69,72^ The hydrophilicity^73^ and rigidity^69,72^ of LbL films can be tuned by the adjustment of physical–chemical parameters of the film build-up, such as pH and/or ionic strength.

**Fig. 2:**
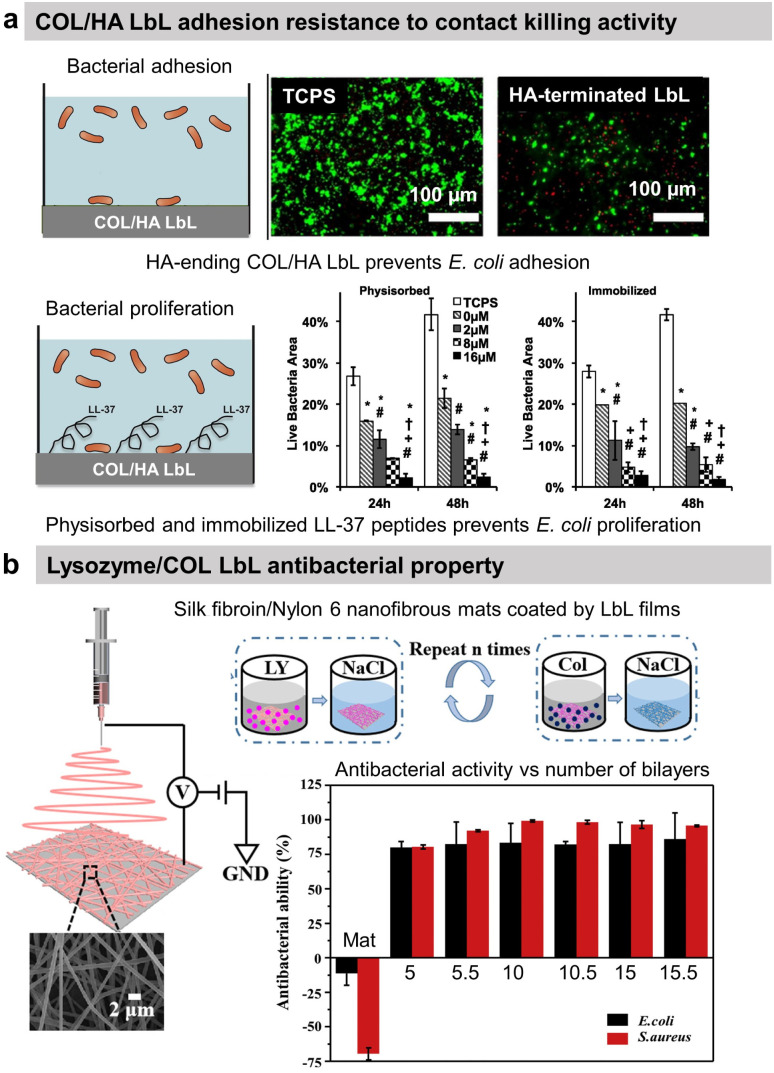
Adhesion-resistant and contact-killing LbL films. (a) Schematic illustration of bacterial tests on native and LL-37 functionalized COL/HA LbL films. Antiadhesive properties of native LbL (0 μM LL-37) and antibacterial activity of LL-37 functionalized LbL against *E. coli* (green = live bacteria and red = dead bacteria). This figure has been adapted from ref. 54 with permission from Elsevier, copyright 2016. (b) Scheme of the fabrication process of silk fibroin/nylon-6 nanofibrous mats by electrospinning and the build-up of COL/lysozyme (Ly) LbL films onto the nanofibrous mats, and the antibacterial activity as a function of the number of bilayers against *S. aureus* and *E. coli*. This figure has been adapted from ref. 87 with permission from Elsevier, copyright 2020.

Regarding protein-based LbL, antiadhesive films were obtained using COL and HA.^54^ After cross-linking of the film using glutaraldehyde, the attachment of *Escherichia coli* (*E. coli*) was decreased by 40% on HA-terminating LbL film compared to uncoated tissue culture plastic substrate (Fig. 2a). No effect on the bacteria adhesion was observed on COL-terminating films. Highlighted by the effect of the ending layer, it was suggested that the HA inhibited the attachment of negatively charged *E. coli* due to the high film hydration and electrostatic repulsion between HA and the bacteria cell wall. Adhesion-resistant films prevent the first step in biofilm formation but fail to kill them, requiring the use of an antibacterial agent.

### Contact-killing films

Antibacterial polymers are positively charged leading to the disruption of the bacteria wall integrity, leakage of the intercellular constituents, and cell death.^74^ Thus, most contact-killing LbL films were developed ended by positively charged polyelectrolytes, either synthetic such as PAH,^75^ quaternary ammonium containing polymers,^76–80^ poly(l-arginine),^81,82^ or poly(l-Lysine)(PLL)^83^ or natural such as chitosan (CHI).^84–86^ Those films are known to avoid any resistance of the bacteria. Other contact-killing strategies were developed using proteins, particularly antibacterial enzymes, or peptides. Lysozyme (Ly) is an antimicrobial enzyme produced by animals able to hydrolyse 1,4-beta-linkages between *N*-acetylmuramic acid and *N*-acetyl-d-glucosamine residues in peptidoglycan, which is the major component of the Gram-positive bacterial wall, hence higher antibacterial activity against *Staphylococcus aureus* (*S. aureus*) strain. Silk fibroin and nylon-6 electrospun nanofibrous mats were functionalized by 10 bilayers of COL/Ly LbL to obtain over 98% reduction of viable *S. aureus* and 87% for *E. coli*, independently of the ending layer (Fig. 2b).^87^ Interestingly, for 5 bilayers, Ly-ending films showed higher antibacterial activity compared to COL-ending ones (Fig. 2b). Quorum-sensing degrading enzymes, such as acylase or amylase, were immobilized with synthetic polyelectrolytes to suppress the biofilm formation of *Pseudomonas aeruginosa* (*P. aeruginosa*) and *S. aureus*. An antimicrobial peptide, LL-37, was physiosorbed or chemically immobilized on cross-linked COL/HA LbL to obtain contact-killing properties toward *E. coli*.^54^ The concentration of the immobilized peptide played an important role in increasing the contact-killing efficiency reaching 85–90% against *E. coli* after 24 h of contact with the highest concentrations (Fig. 2a). The action of contact-killing LbL films is efficient with time but limited to the vicinity of the functionalized surface.

### Release-killing films

Avoiding the use of excess amounts of antibiotics in the systemic circulation, the release-killing films are capable of neutralizing bacterial loads in their surrounding environment. This strategy is pertinent to prevent bacterial adhesion and proliferation during the post-implantation period lasting 6–12 h up to a few days. It has been demonstrated that this period is crucial for the bio(integration) of the biomaterial.^88^ LbL films releasing antimicrobial agents were developed usually *via* (i) the degradation/dissolution of the LbL film or (ii) diffusion from the films.

Antibiotics were loaded in LbL films and the amount was tuned by varying the assembly pH, the incubation time, and the number of deposited layers, and also by using heat treatment.^89^ The degradation of synthetic LbL films, containing antibiotics, was obtained using the hydrolytic degradation of one of the synthesized polyelectrolytes,^90^ by pH changes,^91^ or by an electric potential.^92^ In the last two cases, the charge of one of the components was neutralized leading to the dissolution and release of the antibiotics.

Due to solubility issues, COL-based LbL films were usually built at acidic pH leading to their total or partial dissolution at physiological pH due to changes in the overall charge. This property was used to obtain the release of an antimicrobial peptide (Tet213). The antimicrobial peptide was chemically grafted on type-IV collagen and assembled into LbL film with HA. The antibacterial property of the LbL films was associated with the sustained release of the peptide in a physiological medium (pH 7.4) following the degradation of LbL.^93^ 58.5% and 56.4% of *Porphyromonas gingivalis* (*P. gingivalis*) and *S. aureus* inhibition of the proliferation were obtained respectively after 24 h of contact with 10 layers of COL-peptide/HA as well as significant prevention of early biofilm formation. CHI-ending (COL/CHI)_10_ LbL films functionalized on silk fibroin/polycaprolactone nanofibrous mat showed excellent killing efficacy of around 88% and 80% against *S. aureus* and *E. coli*, respectively. Around 30–35% killing efficacy comes from one CHI layer with an increase of efficiency with the CHI number of layers.^94^ The activity can be associated with CHI release in the surrounding bacteria probably due to the degradation of COL/CHI LbL film. The same LbL film was deposited on polycaprolactone/nylon 6 mats to reach 95% inhibition of *E. coli* and *S. aureus* proliferation.^95^

**Fig. 3:**
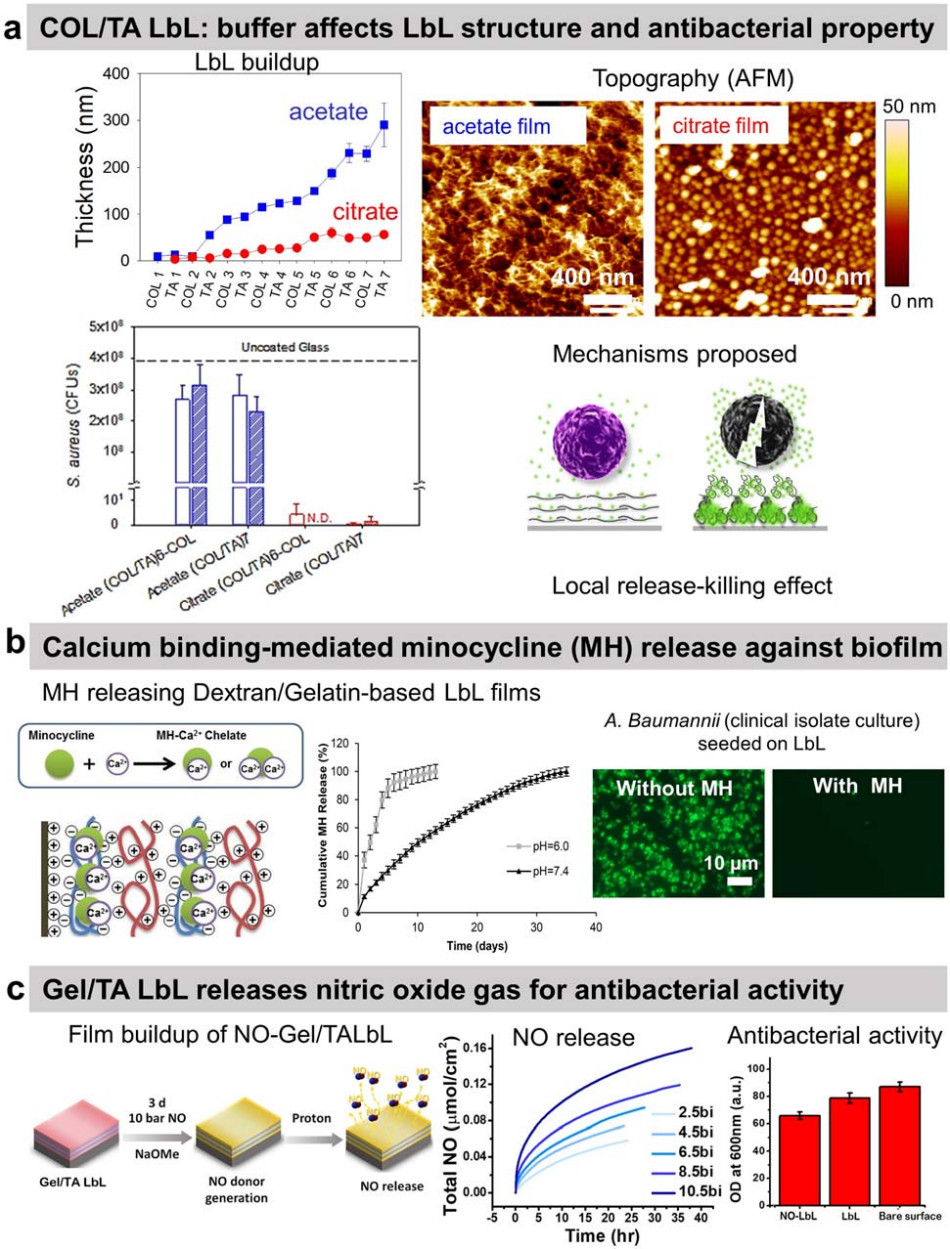
Release-killing LbL films. (a) Effect of the buffer on the build-up, topography, complexation, and antibacterial property of collagen/tannic acid (COL/TA) LbL films. This figure has been adapted from ref. 96 with permission from American Chemical Society, copyright 2010. (b) Ca^2+^-binding mediated sustained release of minocycline (MN) from dextran sulfate/gelatin type A (DS/Gel) LbL shows antibacterial activity. This figure has been adapted from ref. 100 with permission from PLOS ONE, copyright 2014. (c) The release of nitric oxide (NO) gas from gelatin/tannic acid (Gel/TA) LbL film shows antibacterial activity towards *S. aureus*. LbL and NO-LbL stand for Gel/TA and NO-generating moieties functionalized Gel/TA, respectively. This figure has been adapted from ref. 101 with permission from Nature Springer, copyright 2019.

Tannic acid (TA), a polyphenol known for its antibacterial properties, was associated with COL to obtain a release-killing antibacterial effect towards *S. aureus.*^96^ Our group showed the effect of the buffer on the build-up of COL/TA LbL films leading to a dramatic effect on their antibacterial properties. On the contrary to COL/TA acetate films, the granular topography of COL/TA citrate films led to a local release of TA preventing *S. aureus* proliferation for 24 h (Fig. 3a). Nisin, an antimicrobial peptide extensively used in the food industry, was immobilized with poly(acrylic acid) to obtain a release-killing effect thanks to the dissolution of the film.

The diffusion of the antibacterial agent from LbL films was first obtained by immobilizing silver nanoparticles (AgNPs) allowing the release of Ag^+^ ions. Indeed, AgNP's antibacterial properties come from its dissociation in silver ions, which bind to the microbial wall, diffuse into the cell, and interact with proteins/enzymes/DNA.^97^ AgNPs were mostly immobilized in synthetic polyelectrolyte-based LbL by direct incorporation^98^ or reduction of Ag^+^-loaded films leading to an efficient decrease of the bacteria proliferation.^98,99^ Avoiding the use of AgNPs, the diffusion of silver ions was obtained from liposomes containing AgNO_3_ salt embedded in PLL/HA films and using the temperature as a trigger. Exponentially growing films based on polypeptides were exploited to obtain a release-killing effect. The contact with negatively charged pathogens led to the diffusion of polycations towards the interface leading to the death of bacteria.^81–83^

Reduced extracellular pH (tissue acidosis) is common in the case of tissue injury, inflammation, or infection. Thus, the pH-triggered release could be pertinent at the implant–tissue interface. Minocycline hydrochloride (MH), an antibiotic with anti-inflammatory and antibacterial properties, was incorporated into Gel/dextran sulphate (DS) LbL films thanks to the ability of MH and the polymers to form chelates with Ca^2+^ ions (Fig. 3b).^100^ (DS + Ca^2+^/MH + Ca^2+^/Gel + Ca^2+^)_8_ LbL films released the antibiotic for 13 days at pH 6. This release may be due to the weaker chelation between Ca^2+^ ions and DS at lower pH. The coatings prevented bacterial proliferation and biofilm formation of seven pathogenic strains as well as clinical isolate bacteria. The films showed no cytotoxicity towards NIH3T3 mouse fibroblasts. MH released inhibited the production of nitric oxide (NO) from lipopolysaccharide-treated RAW264.7 murine macrophages, thus showing anti-inflammatory potential.

The use of proteins allows to design of gas-release films. Cellobiose dehydrogenase was embedded in zwitterionic polycation/PSS LbL. In the presence of cellobiose, this enzyme produced H_2_O_2_ leading to an antibiofilm effect, *i.e.* a reduction of 53% of *S. aureus* biofilm in comparison to uncoated PDMS catheter.^102^ Gel/TA LbL films were designed to graft, on the secondary amines of Gel, *N*-diazeniumdiolates, and NO-generating moieties (Fig. 3c).^101^ The developed Gel/TA LbL films released NO gas reducing *S. aureus* planktonic growth by 35% with no cytotoxicity towards human dermal fibroblasts. The amount of NO release was controlled by optimizing the film thickness, *i.e.*, the number of bilayers. In addition, thanks to the rough porous structure and high surface area of the LbL film, NO release showed a burst release pattern initially (good for the antibacterial property) followed later by a sustained release (suitable for cell signalling).

## LbL films modulate mammalian cell adhesion

**Scheme 3:**
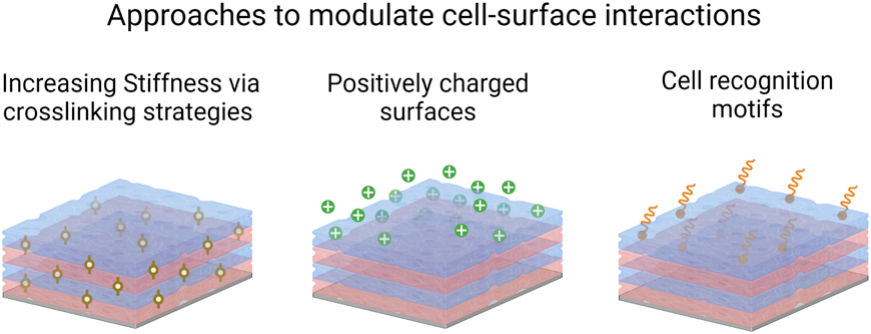
Schematic representation of the LbL strategies to promote cell adhesion.^103^

A primary function of biomaterials is to repair, conserve, or promote a tissue function or an entire organ. Much attention has been devoted to designing cell–biomaterial interfaces, where cells meet the biomaterials. Hence, the surface properties become important to modulate inflammatory cell activation along with mammalian cell adhesion, proliferation, and subsequently differentiation to ensure successful bio-integration. Various strategies were used based on LbL films such as tuning their mechanical properties by ionic or covalent cross-linking and the surface chemistry by introducing positive charges or cell recognition molecules (Scheme 3). LbL films based on polyelectrolytes to modulate cell-surface interactions have been extensively reviewed elsewhere.^103,104^

Synthetic polyelectrolytes can have intrinsic properties, such as PSS, favouring hepatocyte adhesion^30^ progenitor endothelial cells maturation^31^ or the differentiation of myoblasts into myotubes.^105^ Although the underlying mechanism is not understood, we can hypothesis that PSS property could be due to its heparin-like structure. PSS can interact with fibroblast growth factor-2 (FGF2), which plays a role in a range of biological functions such as wound healing, angiogenesis, and bone regeneration. However, to enhance the cell adhesion of primary cells, most synthetic films have to be functionalized by adhesive moieties, such as RGD (arginine–glycine–aspartic) peptide^106^ through the modification of one of the polyelectrolytes. RGD sequence makes up an anchoring place for both α and β integrin sub-units, which enhances the adhesion and proliferation of many kinds of eukaryotic cells. The presence of RGD on top of the films improved the short-term adhesion of primary osteoblasts. Mannose is a common component of lectin, present at the surface of cell membranes, and playing a role in the recognition within the immune system. Mannose-grafted films allowed primary chondrocyte adhesion and proliferation while preventing chondrosarcoma cell growth.^107^ COL, Gel, Fn and ELP have been mostly used to promote cell adhesion by recognition of pro-adhesive moieties through RGD sequence, naturally present in these proteins. Table 1 summarizes protein-based LbL films developed to favour cell adhesion.

**Table 1:** Protein-based LbL films reported to modulate mammalian cell response^a^

LbL system	Type of cells	Ref.
**COL-based LbL films**		
Native/succinylated, maleylated, and citraconylated COL	Murine fibroblasts	116
COL-coated AuNPs/PLL *vs.* COL/PLL	Murine fibroblasts	117
COL-coated AuNPs/photosensitizer-PLL	Murine fibroblasts	118
COL/HA free-standing films	Murine fibroblasts	109
COL/CS *vs.* COL/HA	Murine fibroblasts	112
COL/CHI	Human dermal fibroblasts	115
COL/HA	Macrophage-like cells and *in vivo* rat model	119
COL/HA	Chondrosarcoma	120
COL/Alg	Human periodontal ligament-derived cells	121
COL/Alg	Human umbilical vein endothelial cells, murine fibroblasts and astrocytes	55
		56
COL/lumican	Hepatic stellate cells and murine myoblasts	122
COL/PSS	Murine myoblasts	108
COL/PAA	Murine myoblasts	123
COL/TA	Human myoblasts	124
Methylated/succinylated COL	Hepatocytes	125

**Gel-based LbL films with**		
PDADMA, CHI	Murine fibroblast	126
Gel/laminin, Gel/DS (and COL IV) *vs.* BSA based films	Murine embryonic stem cells	127
Gel/PAH patterned LbL	Murine aorta smooth muscle cells	128
Gel monolayer on PSS/PAH LbL		129
Gel/PSS	Chondrocytes	130

**Fn-based LbL films with**		
Fn monolayer on PSS/PAH LbL	Murine aorta smooth muscle cells	129
Fn monolayer on patterned LbL		128

**ELP-based LbL films with**		
ELP^+^/ELP^−^	Physico-chemical study	131
ELP-PEI/ELP-PAA	Cell adhesion	132

a COL = type I collagen, PSS = poly(styrene sulfonate), HA = hyaluronic acid, DS = dextran sulfate, CS = chondroitin sulfate, CHI = chitosan, Alg = alginate, PAA = poly(acrylic acid), PLL = poly l-lysine, Gel = gelatin, TA = tannic acid, PDADMA = poly(diallyldimethylammonium chloride), BSA = bovine serum albumin, PAH = poly(allylamine hydrochloride), Fn = fibronectin, ELP = elastin, PEI = poly(ethylene imine).

### Collagen-based films

Nicholas A. Kotov and co-workers reported the first COL-based LbL film with PSS to improve the adhesion of C2C12 mouse myoblast on COL-terminating LbL films.^64^ Since then, COL has predominantly been used with various counterparts such as ECM components, polysaccharides, and inorganic micro- to nano-particles in LbL assembly. In general, the films have a fibrillar topography due to the tropocollagen triple helix structure. Using HA as a partner was logical as the polysaccharide is one of the main components of the ECM. Fibrillar COL/HA LbL films showed terminating-layer dependent chondrosarcoma cell adhesion property. Cells seeded on COL-ended films were able to synthesize ECM components.^108^ No cellular matrix was observed on HA-ending films which was attributed to the high water content of HA and the repulsion between the film and the HA produced by the cells at their surface. In this work, the instability of COL/HA films in physiological conditions at room temperature was not addressed, which was put in evidence later.^9^ T. Fujie *et al.* developed freestanding COL/HA LbL films, called nanosheet, using a supporting film method, *i.e.*, the deposition of a sacrificial poly(vinyl alcohol) layer on the substrate before the LbL build-up.^109^ They showed that the incubation of the nanosheet in physiological conditions at 37 °C led to COL fibrinogenesis and HA release, which enhanced both the mechanical stiffness of the surface and adhesive elongation of fibroblasts in contrast to the native nanosheet. The authors suggested that the increased stiffness due to long COL fibrils favoured the formation of the focal adhesion complex and the removal of HA-related repulsive interactions towards the cells improved ligand–receptor binding between COL and integrins. COL/HA LbL films were used to improve the adhesion of pre-osteoblasts and human gingival fibroblasts on titanium disks^110^ and osteoblasts on PLLA substrates.^111^ Native COL/HA LbL films were used *in vivo* to cope with the foreign body response^112^ and cross-linked ones to promote osseointegration.^113^ When implanted in the back of a rat model, HA-ended COL/HA LbL films decreased the thickness of fibrosis by 29–57% compared to uncoated PDMS with only a few macrophage aggregates observed close to implant–tissue interfaces.^114^

**Fig. 4:**
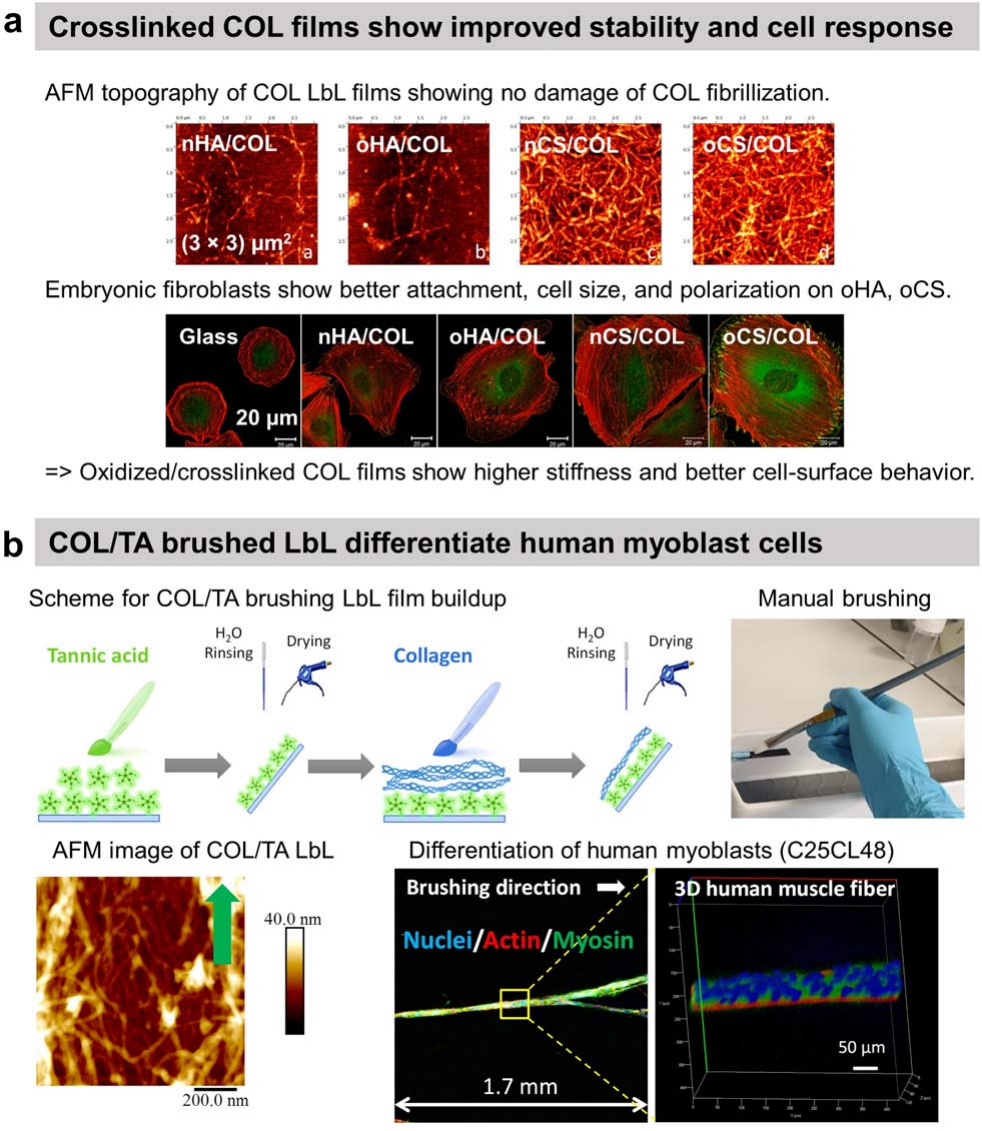
Collagen-based LbL films. (a) Intrinsically crosslinked collagen-based LbL films with oxidized glycosaminoglycans (either hyaluronic acid or chondroitin sulfate) show preservation of COL fibrillar structures and enhanced adhesion, cell size, and polarization of embryonic fibroblasts, thanks to increased film stiffness due to the crosslinking. This figure has been adapted from ref. 115 with permission from American Chemical Society, copyright 2014. (b) Scheme for build-up of oriented collagen/tannic acid (COL/TA) LbL film using a brushing LbL method, and use of the LbL for orientation and differentiation of human myoblasts into myotubes. This figure has been adapted from ref. 124 with permission from American Chemical Society, copyright 2022.

To avoid the antiadhesive effect of HA, COL was assembled into LbL films using chondroitin sulphate (CS). COL-ending COL/CS films showed satisfactory spreading of murine embryonic fibroblasts with higher adhesion, cell density, and area than COL/HA films (Fig. 4a).^115^ This was attributed to the presence of a higher amount of COL fibres and a higher contact area in COL/CS films than in COL/HA films (>20 μg cm^−2^*vs.* 10 μg cm^−2^, respectively). The cell attachment was improved using oxidized CS (oCS) which led to cross-linked and more rigid COL-based LbL films.

Alginate (Alg), a polysaccharide extracted from algae, was associated with COL in LbL films and subsequently cross-linked by carbodiimide chemistry,^121^ glutaraldehyde or genipin^55,56^ to stabilize the film in physiological conditions for human periodontal ligament cells,^121^ endothelial cells,^55^ astrocytes and human gingival fibroblasts culture.^56^ In particular, the two latter cells were aligned on COL-oriented fibres obtained by mechanical stretching of the COL/ALG functionalized PDMS substrates.

COL is an amphoteric macromolecule and thus was also associated with polycations such as CHI^120^ and PLL. Deposited on poly(caprolactone)/cellulose acetate electrospun nanofibrous mat, COL/CHI LbL films improved the migration of human dermal fibroblasts *in vitro* and promoted skin regeneration *in vivo*.^120^ Thanks to the increasing number of COL layers, higher binding site densities were exposed for cells to attach on the surface with a positive effect in neovascularization.

Native COL was also associated with chemically modified COL prepared as deamidated, succinylated, maleylated, and citraconylated derivatives to coat polyacrylonitrile and poly(dl-lactide-*co*-glycolide) fibres^116^ or onto hepatocyte cell layers in a microfluidic setup.^125^ The resulting COL LbL films showed good attachment and spreading of murine fibroblasts. Other properties have been added to COL-based films thanks to the partner. M. K. Saums *et al.* associated COL with lumican, a small leucine-rich proteoglycan involved in the modulation of cell proliferation and differentiation. A better attachment and differentiation of hepatic stellate cells was obtained on COL/lumican than on COL/poly(glutamic acid) films. The fibrous network of COL and the presence of lumican favoured cell differentiation into myofibroblastic phenotype.^122^

COL was also used to coat gold nanoparticles (NPs) improving the fibroblast's adhesion in comparison to COL/PLL thanks to the film rigidity.^117^ By using photosensitizer-coupled PLL combined with COL-coated NPs, the cells were selectively detached from the LbL-coated substrate by irradiating with a laser. Reactive oxygen species were produced by the photosensitizer leading to cell death and detachment.^118^ COL-based films were used to improve the cytocompatibility of quantum dots (QDs) NPs-based LbL.^119^ Used for diagnosis and therapeutical applications but highly toxic, semiconductor QDs-based LbL were coated by COL/polyacrylic acid LbL to alleviate their cytotoxic nature towards C2C12 myoblasts thanks to the deceleration of QDs decomposition.^119^ Recently, our group reported the use of commercially available cheap nylon paintbrushes to build linearly growing COL/TA LbL films. The brushing method allowed the deposition of aligned COL layers, thanks to the shearing effect of the brushing process. The orientation of COL and release of TA from the LbL film led to the alignment and differentiation of human myoblasts into myotubes opening the route of the development of 3D human muscle fibres *in vitro* (Fig. 4b).^124^

### Gelatin-based films

**Fig. 5:**
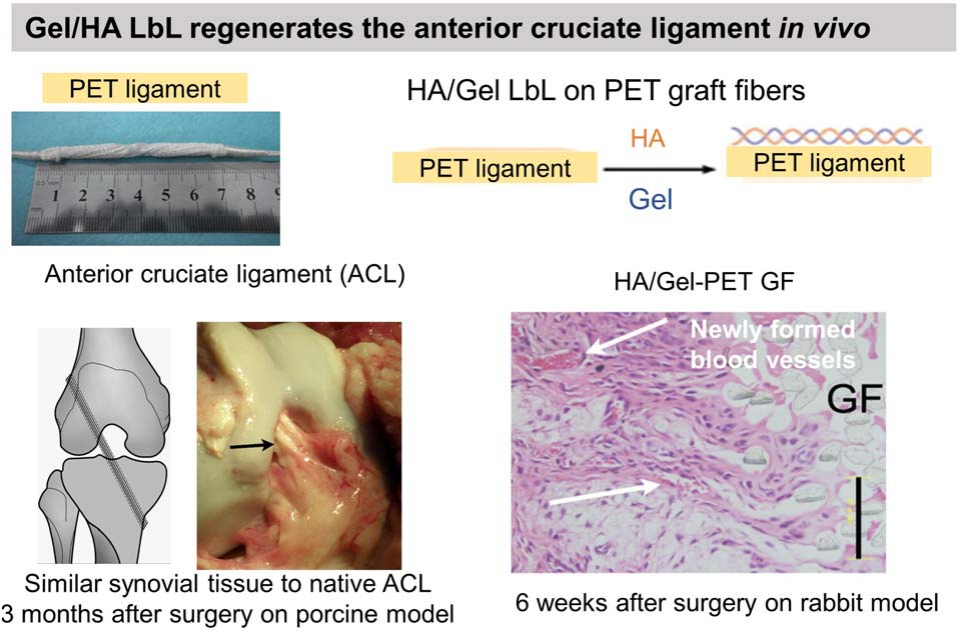
Gelatin and fibronectin-based LbL films. Functionalization of polyethylene terephthalate (PET) ligaments with HA/Gel LbL film implanted *in vivo* in a porcine model showed regeneration of anterior cruciate ligament. This figure has been adapted from ref. 133 with permission from *PLoS One*, copyright 2012.

Gel having an isoelectric point of 5, the assembly pH of Gel/PSS LbL films affected chondrocyte viability.^130^ For assembly at pH 5, the highest chondrocyte viability was observed for only 2 bilayers reaching a plateau for higher bilayers. For assemblies at pH 3 and 7, the chondrocyte viability was observed starting from 4 bilayers. At pH 3 and 7, Gel molecules are highly positively or negatively charged, respectively, which restricts their adsorption in the LbL assembly. At pH 5, a high Gel content was adsorbed with few layers leading to good cytocompatibility. Assembled at pH 6, Gel/CHI LbL films were unstable in physiological pH (7.4) failing to be used for fibroblast culture contrary to Gel/poly(diallyldimethylammonium chloride) (PDADMA).^126^ The pH change induced the deprotonation of the amino groups of CHI leading to a loss of electrostatic interaction between Gel and CHI. Polyethylene terephthalate (PET) engineered ligament grafts, coated with Gel/HA LbL films, significantly suppressed the chronic inflammatory response with the formation of new blood vessels, in the rabbit and porcine model for anterior cruciate ligament reconstruction (Fig. 5a).^133^

### Fibronectin- and laminin-based films

Fn is a glycoprotein known to support cell adhesion, migration, proliferation, and differentiation *via* transmembrane integrin interactions. The protein has been deposited on the top of synthetic polyelectrolyte-based films to promote a faster attachment and further growth of smooth muscle cells in comparison to the native films.^129^ Developed by lithography and lift-off, Gel and Fn-ended LbL patterned surfaces were compared as adhesive proteins with rat aorta smooth muscle cells.^128^ Cells seeded on the synthetic films can migrate toward Fn-ended films leading to good localization of cells on the patterned surface contrary to Gel-ended films.

Having an isoelectric point between 5.5 and 6, Fn was associated with PLL to build LbL films which build-up saturated after 10 bilayers.^134^ It could be due to the interfacial aggregation of Fn leading to the suppression of the available charges on the terminal layer required for successive LbL growth. It was found that Fn can also be associated with PSS at pH 5.8 with an efficient build-up with overnight incubation of the protein.^128^ It was not clear if the adsorption of Fn was electrostatic or due to some other attractive force. E. Brynda *et al.* showed that type IV collagen and Gel-based films associated with DS or laminin, a glycoprotein from the ECM, led to better murine embryonic stem cell attachment in comparison to BSA-based LbL films.^127^

### Elastin-based films

**Fig. 6:**
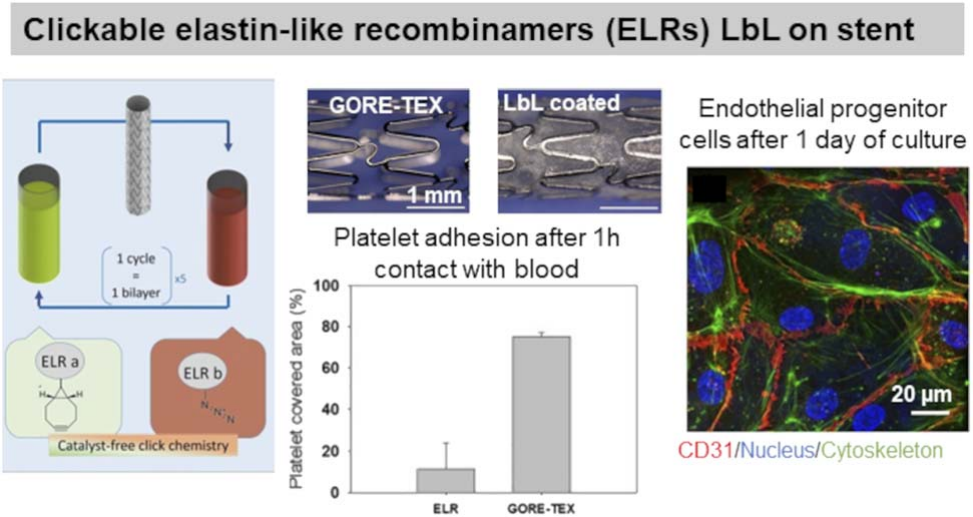
Use of elastin in LbL films. Clickable elastin-like recombinamers (ELR), modified by either azide or cyclooctyne, LbL film on coronary stent reduces platelet adhesion and promotes endothelial layer formation, thanks to the presence of RGD-sequence on ELR-azide. This figure has been adapted from ref. 136 with permission from Elsevier, copyright 2019.

ELP, a fibrous ECM protein composed of single tropoelastin subunits, presents repeating amino acids sequence VPGVP (V: valine, P: proline, G: glycine). The cell-ELP interactions are attributed to elastin receptors, G protein-coupled receptors, and integrins.^135^ Elastin-like polypeptides have amino acid sequences derived from tropoelastin consisting of repeating units of a pentapeptide VPGXG, where X can be any amino acid except for proline. Obtained by recombinant DNA techniques, they are produced by bacteria cells and are called also elastin-like recombinamers (ELR). They exhibit a reversible phase-transition in aqueous solution and are soluble below the transition temperature (*T*_t_) and phase-separate into coacervates above *T*_t,_ which is mostly 37 °C. Thermoresponsive LbL films were prepared using ionic ELP containing lysine units (polycation) and glutamic acid units (polyanion). Interestingly, long exposure (72 h) of the films with a salt solution at 37 °C (higher than *T*_t_) caused the film to shrink, thus improving its stability.^131^ Clickable ELR LbL films were developed to favour endothelialisation of stents withstanding high shear stress flow, limiting platelet adhesion and blood coagulation. The LbL films led to a confluent layer of endothelial progenitor cells after 1 day of culture, thanks to the presence of the RGD sequence (Fig. 6).^136^ ELR-based LbL films were built using poly(ethylene imine)-ELP and polyacrylic acid-ELP, obtained by chemical coupling, to favour fibroblasts' focal adhesion point.^132^

## Designing hemocompatible surfaces

**Scheme 4:**
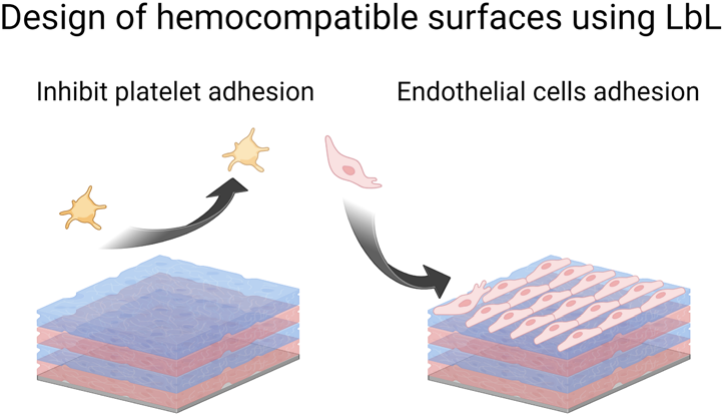
Schematic illustration of LbL strategies to design hemocompatible surfaces.

Blood circulation ensures the supply of all tissues with oxygen, and nutrients, and the removal of metabolites. It is ensured by a balance of pro and anti-coagulant factors in the vessels. The presence of a biomedical device disturbs this balance leading to thrombosis and infarctions or inducing bleeding. As soon as blood or plasma contacts a foreign surface, haemostasis, and blood clotting occur during the first few milliseconds. Besides being a healthy response by the body, haemostasis can block blood flow in blood vessels. To avoid this situation, anticoagulant drugs are given but they could be associated with enhanced bleeding risks. Surface functionalization of implants by LbL films offers various routes to prevent platelet adhesion (Scheme 4) and then blood coagulation. Moreover, LbL can also favour the colonization of endothelial cells, forming the inner layer of blood vessels and modulating antithrombotic factors.

### LbL films can prevent platelet adhesion

To suppress blood activation processes, highly hydrophilic films provide a stealth effect on the surface, which should prevent the interaction with cells and proteins in the blood.

**Fig. 7:**
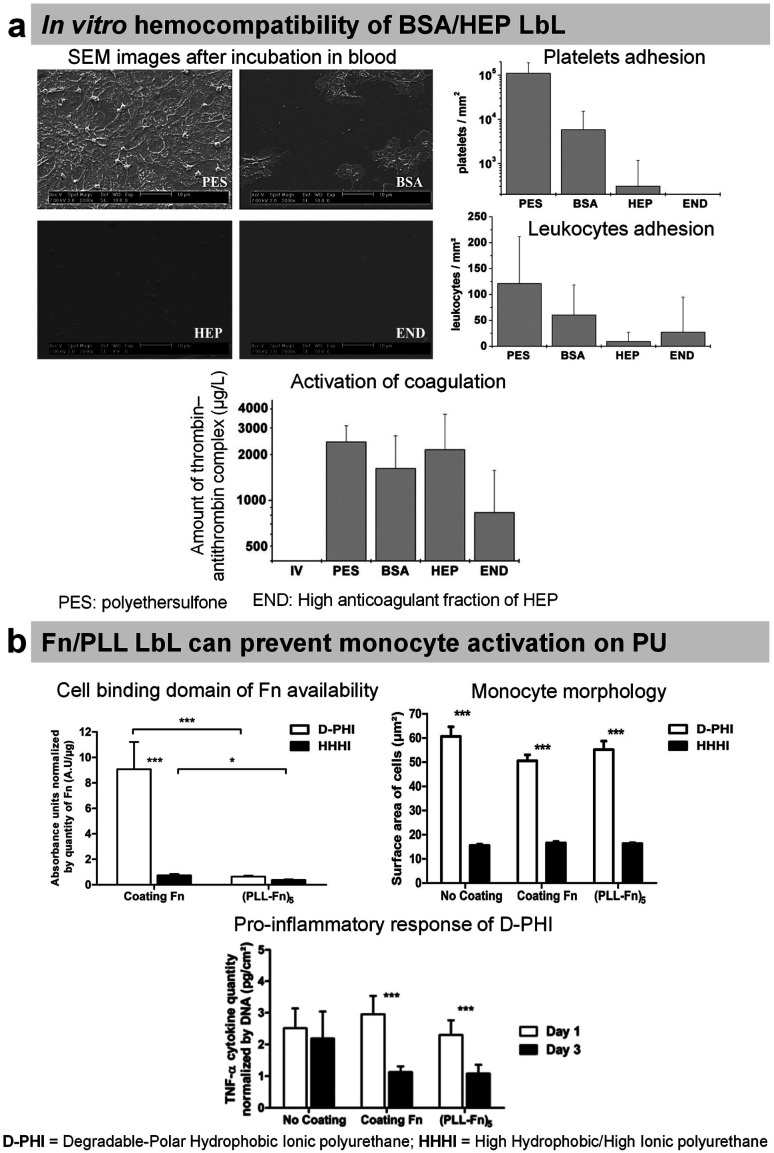
LbL films inhibit platelet adhesion. (a) Bovine serum albumin/heparin (BSA/HEP) LbL on polyethersulfone foils (PES) reduces the adhesion of platelets and leukocytes upon incubation in slightly heparinized (1.5 IU heparin per mL) human whole blood *in vitro*. This figure has been adapted from ref. 146 with permission from John Wiley and Sons, copyright 2006. (b) (Fn/PLL) LbL films deposited on different polyurethanes influence the behavior of monocytes. This figure has been adapted from ref. 149 with permission from Elsevier, copyright 2018.

Synthetic LbL films were used to decrease the protein adsorption leading to the suppression of platelet adhesion. Usually, heparin-like,^137^ zwitterionic,^138^ phosphorylcholine,^139,140^ or poly(ethylene glycol) based LbL^141^ were used for this purpose requiring chemical synthesis. Protein-based LbL films were used to functionalize diverse types of surfaces using bovin serum albumin (BSA), a plasma protein with anti-thrombogenic properties, to reduce non-specific platelet adhesion. Jian Ji *et al.* assembled BSA/PEI LbL films on poly(vinyl chloride) (PVC)^142^ and 316 L stainless steel (SS)^143^ surfaces. Built at around physiological pH, the films were stable for up to 45 days in physiological medium under static conditions with less than 10% BSA released. On 4 BSA/PEI bilayers-coated PVC surfaces, platelet adhesion was negligible. Whereas on 316L SS, due to granular topography, the homogeneous surface coverage was obtained after 8 bilayers which reduced the platelet adhesion by 90%. Similarly, sulphated polysaccharide heparin (HEP), a commercially known anticoagulant drug, was incorporated into LbL films with BSA,^144–146^ streptavidin,^147^ Fn,^148,149^ and COL.^150,151^ BSA/HEP LbL films were used to functionalize PVC,^144^ polystyrene (PS) surfaces^145^ and poly(ether sulfone) (PES) foils.^146^ Built at pH 3.9 on PVC sheets, BSA/HEP films were unstable in PBS due to the reversal charge of BSA (pI 4.9) at physiological pH. After cross-linking by glutaraldehyde, HEP-ending BSA/HEP films suppressed platelet aggregation and decreased platelet adhesion in comparison to untreated PVC substrate. Higher anticoagulant efficiency of uncross-linked films was attributed to the release of HEP.^144^ On PS substrates, BSA/HEP films built at pH 4 and cross-linked by glutaraldehyde showed a reduced platelet adhesion with the increase in bilayers. This result was similar with only BSA cross-linked LbL films. Dried and reswollen (BSA/HEP)_3_ coatings showed a moderate platelet attachment. HEP once incorporated into LbL films loses its thrombin inhibition by less than 10%, perhaps due to HEP interactions within LbL assembly. Thrombin inhibition efficacy was also improved by increasing the number of bilayers.^145^ BSA/HEP multilayers with two kinds of HEP (standard, and high anticoagulant fraction) were built at pH 4 on PES foils. Interestingly, the type of HEP solely affected the biological properties of LbL films. Platelet adhesion was roughly comparable on BSA/HEP and BSA control coatings. However, BSA/HEP reduced the coagulation activation especially when using a high anticoagulant fraction of HEP (Fig. 7a). Such coatings could be used in blood purification systems.^146^

Heparinylated multilayers were constructed using streptavidin/biotin bio-specific interaction on titanium. The films showed reduced platelet adhesion and enhanced clotting time.^147^ Fn is known to interact with leukocytes. Fn/PLL LbL films were deposited on hydrophobic polyurethane to study the protein conformation and the response of monocytes. Fn adsorbed in unfolded state in Fn/PLL LbL, due to the negatively charged cell binding domain (RGD domain) and sub-domains with the C-terminal of Fn interacting with positively charged PLL. This leads to the low availability of Fn cell binding domain suppressing monocyte activation (Fig. 7b).^149^ These films alleviated the pro-inflammatory response of monocytes in contact with degradable polar hydrophobic polyurethane for 3 days.

Titanium was modified by COL/HEP using electrostatic LbL assembly. The coated surfaces show decreased platelet adhesion and activation, and an increment in the clotting time.^150,151^ Oxidized dopamine (oDOP) used as precursor layer on titanium allowed obtaining COL/HEP LbL films with higher thickness, prolonged thromboplastin activation time, lower haemolysis ratios, and platelet coverage.^152^ COL/HEP coatings were compared with DOP/HEP. Though DOP/HEP showed a better adhesion of the coating and stability of the film, COL/HEP is better at maintaining anti-coagulant efficiency.^153^ Yet, the film's stability over a prolonged time remains a challenge.

### LbL films can promote endothelial cell adhesion

Endothelial cells (ECs) form the inner layer of blood and lymphatic vessels. ECs possess anti-thrombogenic potential by maintaining a physiological barrier between outer host tissue and circulating blood. An incomplete endothelialisation can cause other damages like restenosis and neointimal hyperplasia. Rapid endothelialisation is considered a better and long-lasting solution for blood-contacting surfaces.^154–156^ PSS/PAH LbL was reported to favour endothelial cell adhesion and proliferation with more spreading cells on polycation-terminated films than on polyanion-terminated films without disturbing the adhesion mechanism.^157–160^ Deposited in the inner part of arteries, PSS/PAH films showed good *in vivo* retention of the luminal surface.^161^ PAH/PSS LbL film improves also the EPC's maturation into functional and mature ECs.^31^ This could be related to the heparin-like properties of PSS.

**Fig. 8:**
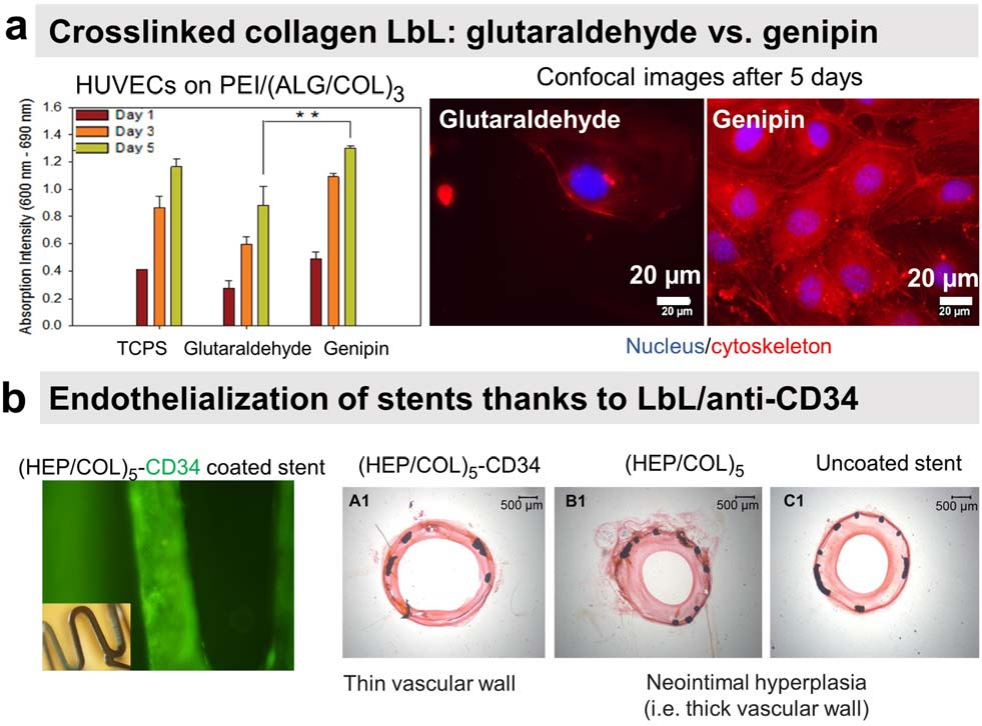
LbL films promote endothelial cell adhesion. (a) Human umbilical vein endothelial cells (HUVECs) formed a confluent layer on genipin cross-linked (ALG/COL) LbL after 5 days compared to glutaraldehyde ones. This figure has been adapted from ref. 55 with permission from American Chemical Society, copyright 2012. (b) Collagen/heparin (COL/HEP) LbL ended by CD34 antibody coated on stents showed rapid endothelialisation and no neointimal hyperplasia in rabbit femoral arteries *in vivo*. This figure has been adapted from ref. 166 with permission from Elsevier, copyright 2010.

Our group investigated the effect of chemical cross-linking on the endothelialisation of COL/ALG LbL films. On the contrary to glutaraldehyde, genipin, a natural plant-derived agent, cross-linked coatings showed rapid attachment of human vascular ECs within 1 h, leading to a confluent layer in 5 days (Fig. 8a).^55^ Combining the benefits of COL biocompatibility and HEP anticoagulant capacity, COL/HEP coatings on intravascular stents showed antithrombic properties with good adhesion and proliferation towards human umbilical vein endothelial cells (HUVECs).^154^*In vivo* assessments were performed to better elucidate the vascularization potential of multilayer films. Better angiogenesis was obtained with COL/HEP coated porous hydroxyapatite scaffolds with enhanced mechanical properties using the chicken chorioallantois membrane model.^155^ COL IV/HEP modified cardiovascular titanium stent surfaces exhibited new angiogenesis after 15 days in dog femoral artery model.^156^ Another perspective within the vascularization approach is to develop multifunctional coatings with selectivity towards ECs to support early and fast endothelialisation. For this, COL/HEP LbL films were immobilized REDV peptide, recognized by ECs. Hydrophobic, Teflon® (ePTFE) films were coated with COL/HEP films and showed weak platelet activation and adhesion, prolonged coagulation time, and reduced haemolysis. REDV-containing films showed enhanced early cell attachment, proliferation (cell density after 72 h), and cell activity.^162^ The delivery of growth factor to the host site can potentially enhance endothelialisation and angiogenesis. However, their controlled delivery is challenging due to the extremely small half-life of growth factors (less than 1 hour) and susceptibility to degradation in a physiological environment or under the action of enzymes.^153^ Their embedding into LbL films could overcome this issue.^163^ Prolonged release (above 35 days) of b-FGF from COL/HEP LbL films showed enhanced angiogenesis of subcutaneous tissue of rat model, *i.e.* high blood vessels' density and diameter.^153^ Antibodies can improve EC specificity by their cell-surface antigen interactions. LbL assembly provides a platform for antibody immobilization to selectively capture EC from the circulating blood for vascular tissue engineering. CD34 is an antigen that specifically presents EPCs. Electrostatically immobilized anti-CD34 into COL/HEP LbL films on Ti surface formed compact EC lining just after 2 days,^164^ compared to 3 days reported for COL/HEP films without the antibody.^165^ Stable under static and flow conditions, COL/HEP LbL films ended by anti-CD34 antibody enhanced *in vitro* early attachment (1 h) of EPCs and significantly reduced neo-intimal formation in rabbit femoral artery model *in vivo* (Fig. 8b).^166^ Commercially available ePTFE grafts were functionalized with COL/HEP LbL loaded with anti-CD 133. CD133 is more specific than CD34 as a surface marker for EPCs. Antibodies functionalized grafts showed enhanced EPCs adhesion and *in situ* rapid early endothelialisation in a porcine carotid artery transplantation model. However, further investigations with smaller diameters (<6 mm) of the grafts and host-relevant antibodies are essential.^167^

## Drug, cytokine, growth factor, and gene delivery

**Scheme 5:**
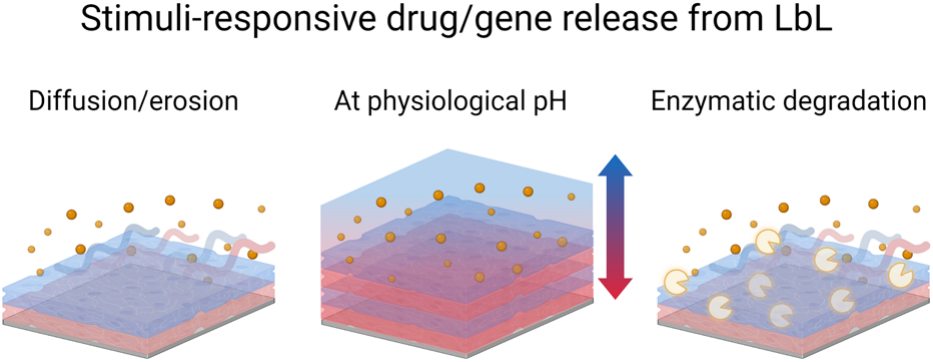
Schematic illustration of drug/gene release from the LbL films under various stimuli *i.e.*, diffusion or erosion, pH change, and enzymatic degradation.

Sustained and controlled release of drug formulations is essential for higher drug efficiency and lower risks of toxicity. Towards this, the LbL technology offers enormous possibilities and is a far better replacement for conventional drug encapsulation techniques.^168^ For instance, the LbL films allow the nanometric control over the order, location, and concentration or loading of various cargo layers like polymer, drug, growth factor, cytokine, or gene.^169^ Park *et al.* reviewed the various methods used for preparing LbL films with drugs.^170^ LbL-assembled films were also used to develop hollow micro–nano capsule payload carriers.^171^ LbL capsules offer unique advantages over conventional carriers (*e.g.* liposomes). The versatility of the LbL method allows for control of the inner cavity of the capsules as well as their surface allowing multi-functionalisation for therapy, diagnosis, or both (*e.g.*, theranostic). A recent review summarizes the recent development of this type of capsule.^172^ In this context, protein-based LbL films were coated onto polystyrene, calcium carbonate, or melamine formaldehyde nano–micro particles to be further dissolved to obtain hollow capsules. The main advantages of using proteins, instead of synthetic polyelectrolytes, are to develop microcapsules with the possibility of obtaining the (i) dissolution in a physiological medium due to a pH change and/or (ii) degradation by enzymes for the drug release (Scheme 5). They are also used to give specific properties to the films or capsules such as controlling mammalian cell fate by using growth factors, plasmids, or recognition by using antibodies. A summary of protein-based LbL drug and gene delivery systems is given in Table 2.

**Table 2:** Protein-based LbL drug and gene delivery systems^a^

LbL system	Trigger	Drug/application	Ref.
**COL-based multilayers**			
Crosslinked COL/sirolimus	Elution	Sirolimus/stents	173

**Gel-based multilayers**			
Gel/EGCG	—	Drug delivery	174
Gel/DS	Ca^2+^, pH	Minocycline delivery	100
Gel/DS, HEP	PBS degradation	Nerve growth factor (NGF), neural application	175
Gel/Alg, Gel/DS	—	Naproxen delivery/anti-inflammatory	176
Gel/PSS nanocapsules	PBS degradation	Lornoxicam delivery	177
Gel/PSS	pH	Furosemide	178
Gel/PDDA capsules	pH	—	179

**Albumins-based multilayers**			
BSA/PDADMA	—	—	180
BSA/IgG	—	—	181
BSA, curcumin-BSA/CHI	pH	Curcumin, doxorubicin/therapeutics	182
BSA/ssDNA	Enzymatic	DNA delivery/gene therapy	183
BSA NPs/CHI	pH	Doxorubicin delivery	184
BSA, pepsin/TA	pH	IgG, β-lactoglobulin/oral delivery	185
BSA/TA	Enzymatic	Drug delivery	186
BSA/polyphenols	pH	IgG, site-selective bioactivity	187
HSA/DMPA	pH	Ibuprofen	188
Ovalbumin/poly-1	Hydrolytic	Transcutaneous drug or vaccine delivery	189

**Protamine sulfate (PRM) based multilayers**			
PRM/DS	—	—	190
PRM/Alg	pH	α-Chymotrypsin	191
PRM/Alg	pH	Anticancer	192
PRM/HA		Anticancer	193
PRM/HEP	pH, trypsin	Anticancer	194
PRM/PSS	pH	Ibuprofen, site-selective bioactivity	195
PRM/CMC	Enzymatic	Doxorubicin/targeted delivery	196
PRM/HGF-pDNA		HGF/transfection	197
PRM/DNA	Enzymatic	Gene delivery	198
Gel/CMC, PRM/DS	pH	EGCG, anticancer	199

**Others**			
Growth factor/DS	Diffusion, erosion	TGF-β1, PDF-ββ, IGF-1/drug or gene delivery, tissue regeneration	200
Growth factor/HEP			
PLL/HA	Diffusion	Interleukin-4/immunomodulation	201 and 202

a COL = type I collagen, Gel = gelatin, EGCG = epigallocatechin gallate, DS = dextran sulfate, HEP: heparin, Alg = alginate, PSS = poly(styrene sulfonate), PDADMA = poly(diallyldimethylammonium chloride), BSA = bovine serum albumin, IgG: immunoglobulin, CHI = chitosan, TA: tannic acid, protamine sulfate: PRM; CMC: carboxymethylcellulose, HGF-pDNA: plasmid DNA encoding hepatocyte growth factor; PLL: poly(l-lysin).

### Release by diffusion/erosion

Once implanted into the human body, immune cells, mainly monocytes and macrophages, are recruited to the implant surfaces within hours post-surgery and initiate the cascades of early inflammatory responses. Although initial inflammation is required for tissue healing, adverse immune reactions in the implanted biomaterials take place, leading to fibrous encapsulation. Immunosuppressive drugs, such as sirolimus, inhibit or decrease the intensity of the immune response. Cross-linked LbL COL coatings were developed to be used as a drug reservoir for the sustained release of sirolimus.^173^ Approved by the Food and Drug Administration (FDA), the drug reduces neo-intimal thickening in models of vascular injury. COL/sirolimus LbL films were built by spray coating and further cross-linked using genipin. From cross-linked COL films, sirolimus was eluted for up to 28 days depending on the number of deposited bilayers.

One of the key components of the inflammatory response is macrophage polarization. The cells can differentiate into pro-inflammatory M1 macrophages, associated with classic signs of inflammation, or anti-inflammatory and pro-healing M2 macrophages, alleviating inflammation and generating a favourable immune microenvironment for tissue healing. A prolonged inflammation due to the accumulation of M1 macrophages gives rise to the formation of fibrous encapsulation, a barrier to implant integration. Cytokines are small cell-signaling proteins secreted by immune cells to help control the inflammation of the body. Thus, LbL films were developed to release Interleukin-4 (IL-4, a M2 polarizing cytokine). In contrast to the uncoated substrates where the monocytes produced high levels of pro-inflammatory cytokines, the released IL-4 from PLL/HA-Aldehyde LbL drives the monocyte differentiation into M2 macrophages over M1 macrophages.^201^ The release of IL-4 from CHI/dermatan sulphate LbL, built on polypropylene mesh, was tuneable based on the number of coating bilayers. IL-4 was detected up to 14, 22, and 30 days for coatings of 20, 40 and 60 bilayers. *In vitro*, macrophage culture assays showed that implants coated with IL-4 promote the polarization towards M2 macrophages, although the concentration of released IL-4 (2.25 ng mL^−1^) was lower than the positive control (20 ng mL^−1^). After 7 days of implantation in mice subcutaneous pocket, the coated polypropylene mesh revealed within the first 50 μm the presence of the greatest amount of M2 macrophages than M1 macrophages *versus* uncoated mesh, suggesting that the effects of IL-4 released from the LbL coating are limited locally. At 90 days post-implantation, IL-4 loaded mesh had a reduced capsule area and thickness compared to the prominent and dense capsules surrounding uncoated mesh.^202^

**Fig. 9:**
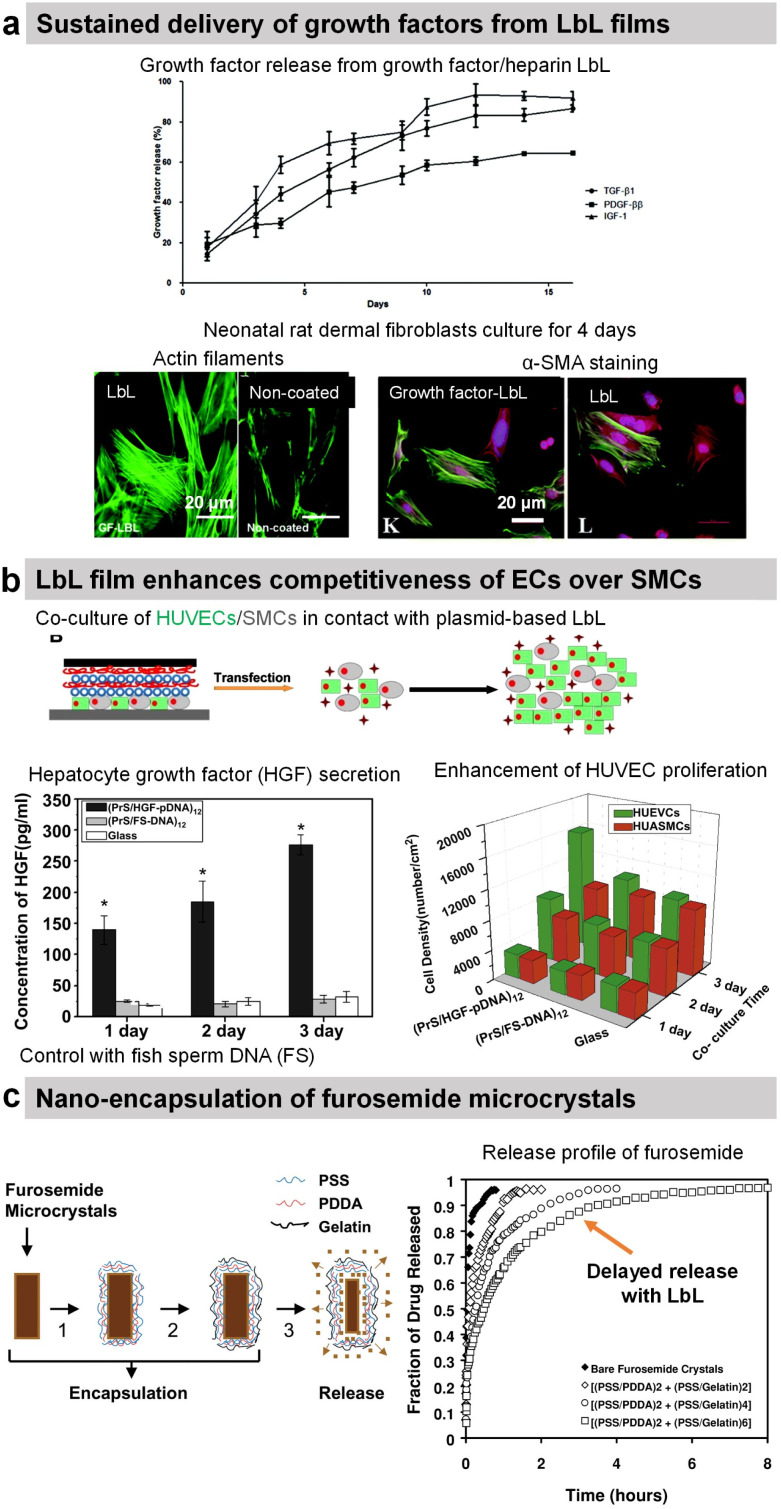
Drug release by diffusion or erosion. (a) Efficient loading and sustained delivery of transforming growth factor beta 1, platelet-derived growth factor ββ, and insulin growth factor 1 from either growth factor/heparin LbL films. The films resulted in the proliferation, migration, and differentiation of fibroblast cells into myofibroblasts (α-SMA labelling). This figure has been adapted from ref. 200 with permission from the Royal Society of Chemistry, copyright 2020. (b) Protamine sulfate/hepatocyte growth factor-pDNA (PRM/HGF-pDNA) LbL, on top of human umbilical vein endothelial cells (HUVEC) and human umbilical artery smooth muscle cells (SMC), induced the secretion of HGF by the cells and showed competitiveness of ECs over SMCs. This figure has been adapted from ref. 197 with permission from Elsevier, copyright 2013. (c) Direct nano-encapsulation of furosemide microcrystals with Gel/PSS LbL to enable sustained drug release profiles. This figure has been adapted from ref. 178 with permission from Elsevier, copyright 2003.

Transforming growth factor-beta 1 (TGF-β1), platelet-derived growth factor ββ (PDGF-ββ), and insulin growth factor 1 (IGF-1) involved in tissue morphogenesis were loaded with the highest loading in combination with HEP or DS. The release of the growth factors was obtained by diffusion and erosion in a controlled manner with the bioactivity maintained for up to 14 days (Fig. 9a).^200^ The activity of growth factors was preserved and allowed increasing fibroblast proliferation as well as enhancing myofibroblast differentiation as put in evidence by α-SMA labelling.

Protamine sulphate (PRM) is a natural arginine-rich protein involved in the condensation of DNA. It is also FDA-approved for the treatment of heparin overdose or excessive bleeding. The LbL-assembled films of protamine and plasmid DNA encoding hepatocyte growth factor were used to promote the growth of HUVECs and hinder the growth of artery smooth muscle cells in a co-culture.^197^ Both cells were transfected upon contact with the plasmid-based LbL film, enhancing the competitiveness of HUVECs over smooth muscle cells (Fig. 9b).^197^ Indeed in comparison to glass and fish sperm DNA (control), the cell density of HUVECs was higher than the cell density of smooth muscle cells after 3 days of culture. Graphene oxide (GO) nanosheets were coated with PRM/Alg LbL film, which not only improved the dispersibility and stability under physiological conditions but also reduced protein adsorption. The LbL-coated GO nanosheets loaded with doxorubicin (Dox) showed better cellular uptake and cytotoxicity against MCF-7 cells.^192^ In comparison with bare Ibuprofen crystals, the uncoated microcapsules showed faster release of Ibuprofen in gastric fluid and slower release in intestinal fluid. The PRM/PSS coating prevented the initial burst release and resulted in sustained release of the drug in both fluids.^195^

The LbL method was used for the direct functionalization of drug-based crystals to serve as a diffusion barrier to control the release of the drug payload. For example, Ibuprofen crystals were coated with HSA/l-α-dimyristoyl phosphatidic acid LbL film for controlled drug release in a solution of pH 7.4. The release rate was controlled by increasing the number of layers and the crystal size.^188^ Furosemide dye microcrystals were coated with Gel/PSS LbL film (thickness around 40–115 nm for 2–6 bilayers) that reduced the release rate of furosemide by 50–300 times in aqueous solutions (Fig. 9c). The media at pH 7.4 showed 6–8 times faster release than the one at pH 1.4.^178^ design of the experiment with aims to enable scale-up production and sustained dissolution (from 120 min to 270 min).^177^ Gel/PSS LbL-coated Naproxen microcrystals, a non-steroidal anti-inflammatory drug, showed a delayed release of the core payload in PBS pH 7.4 (approximately 50% lower dissolution rate).^176^

### Release at physiological pH

**Fig. 10:**
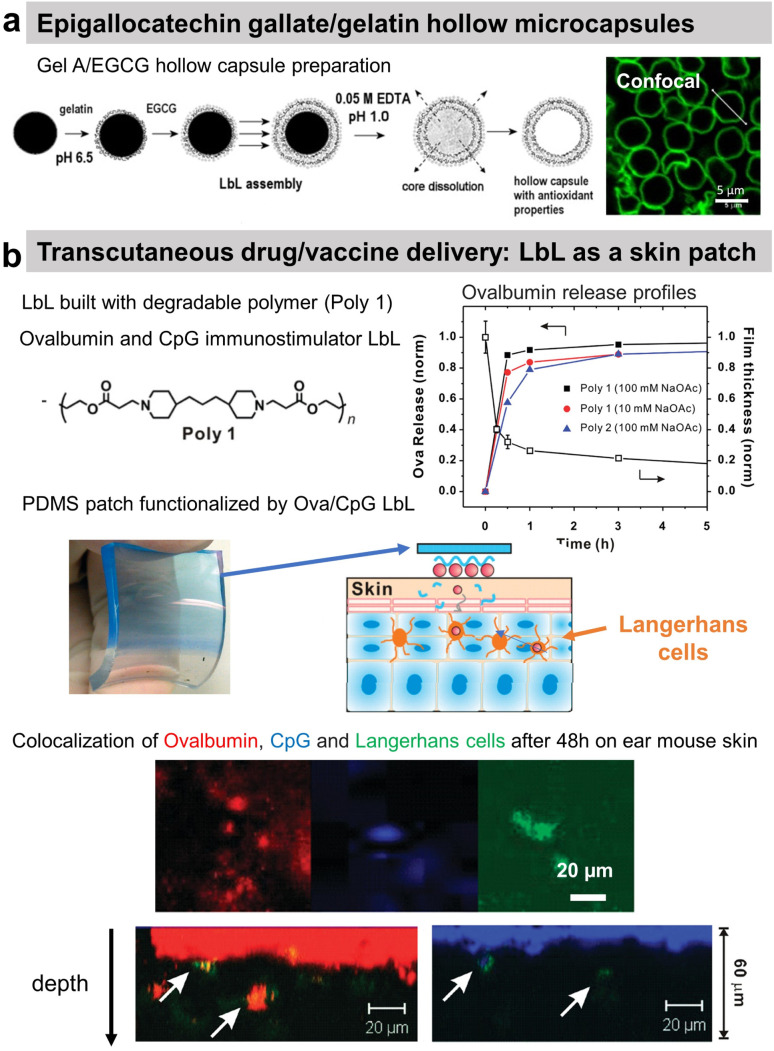
Drug release at physiological pH. (a) Scheme of gelatin A/epigallocatechin gallate (Gel/EGCG) LbL hollow capsule preparation, and fluorescence/AFM images. This figure has been adapted from ref. 174 with permission from Elsevier, copyright 2009. (b) Hydrolysable polymer associated with ovalbumin and CpG, the release of ovalbumin, an optical image of LbL-coated PDMS substrate, and the scheme of its application onto tape-stripped ear skin and confocal images showing penetration of fluorophore-conjugated ova (red) and CpG (blue) released from LbL films into the skin. This figure has been adapted from ref. 189 with permission from American Chemical Society, copyright 2009.

Polyphenols, plant-derived secondary metabolites, possess potentially broad bio-functionalities like antibacterial, antioxidant, and anticancer properties. However, low bioavailability and half-life limit their use as free compounds both *in vitro* and *in vivo*. Hence, a targeted delivery approach with controlled release is necessary. Lvov and co-workers developed Gel/epigallocatechin gallate (EGCG)-based hollow microcapsules with antioxidant properties boosted by increasing the number of bilayers from 1 to 10 (Fig. 10a). Only the permeability of the capsules was tested using dextran of different molecular weights.^174^ Moreover, they developed cross-linked type A Gel nanoparticles (NPs), of around 200 nm diameter using the desolvation method, coated with LbL films, *e.g.*, carboxymethyl cellulose/type A Gel (CMC/Gel)_*n*_. EGCG; a chemopreventive polyphenol, was successfully loaded at pH 4 and subsequently released at pH 7.5 from the nanoparticles to block the HGF-intracellular signalling in the breast cancer (MBA-MD-231) cells. The LbL-coated Gel NPs showed pH-triggered controlled release of EGCG, unlike the burst release within the first 15 min from the uncoated Gel NPs.^199^

A multi-drug carrier, based on BSA/CHI LbL, was designed to deliver both hydrophobic (pyrene and curcumin) and hydrophilic DOX drugs. Microcapsules were built at acidic pH using a mixture of BSA/hydrophobic drug and CHI solutions. The hydrophilic drug was incorporated in a second step by soaking the film. The release of both types of drug was obtained in physiological pH.^182^ BSA nanoparticles/CHI LbL were designed to load and deliver doxorubicin (Dox) under a pH trigger.^184^ A proteolytic enzyme, α-chymotrypsin, was loaded and subsequently released from PRM-based LbL capsules under acidic and above neutral pH, respectively.^191^ Using the hydrolytic nature of a synthetic polymer, the LbL strategy was first reported by Hammond and workers for dual drug delivery in the murine ear skin model *in vivo*. They described the use of a cationic polymer, poly(amino ester) named poly-1, with a protein antigen, ovalbumin (ova), and/or immunostimulatory CpG (cytosine-phosphate-diester-guanine-rich) DNA oligonucleotide adjuvant molecules for LbL assembly. Applied as a skin patch, such dual delivery LbL films released ovalbumin and the CpG in a rapid and sustained manner by hydrolytic degradation (Fig. 10b).^189^*In vivo* experiments showed the penetration of ovalbumin and CpG and colocalization with Langherans cells (cells from the immune system).

### Release by enzymatic degradation

COL/HA LbL hollow microcapsules showed sustained release of fluorescently labelled BSA triggered by collagenase degradation. The release could be controlled by the number of layers, COL cross-linking, and collagenase concentration.^203^ Microcapsules based on BSA/TA hydrogen-bonded LbL assembly were developed to deliver hydrophilic-fluorescently-labelled BSA and hydrophobic 3,4,9,10-tetra-(hectoxy-carbonyl)-perylene, thanks to enzymatic degradation by α-chymotrypsin. The developed capsules were not toxic to murine RAW264.7 macrophages at payload concentrations of around 50 capsules per cell. Such payload carriers may find applications in intravenous drug delivery with an ability for site-specific release.^186^ Moreover, BSA/TA microcapsules were reported to be stable in simulated gastric fluid but degrade in simulated intestinal fluid. Thus, opening possibilities to deliver bioactive compounds and functional foods to the lower gastrointestinal tract. Immunoglobulin G (IgG), a milk protein that can adhere to the human intestinal surface, was incorporated into the LbL film, while β-lactoglobulin could not adsorb. IgG incorporation enhanced the adhesion of the capsules to Caco-2 cells by more than 6 times.^185^ Multiple polyphenols like EGCG, 3,4-*O*-dicaffeoylquinic acid (3,4-diCQA), and TA were alternatingly assembled with BSA to develop robust microcapsules for delivery of IgG in the gastrointestinal tract. Thanks to the synergistic stability due to the use of multiple polyphenols, the capsules, were stable through stomach digestion, showed higher thermal denaturation temperatures, and improved antioxidant potential.^187^

**Fig. 11:**
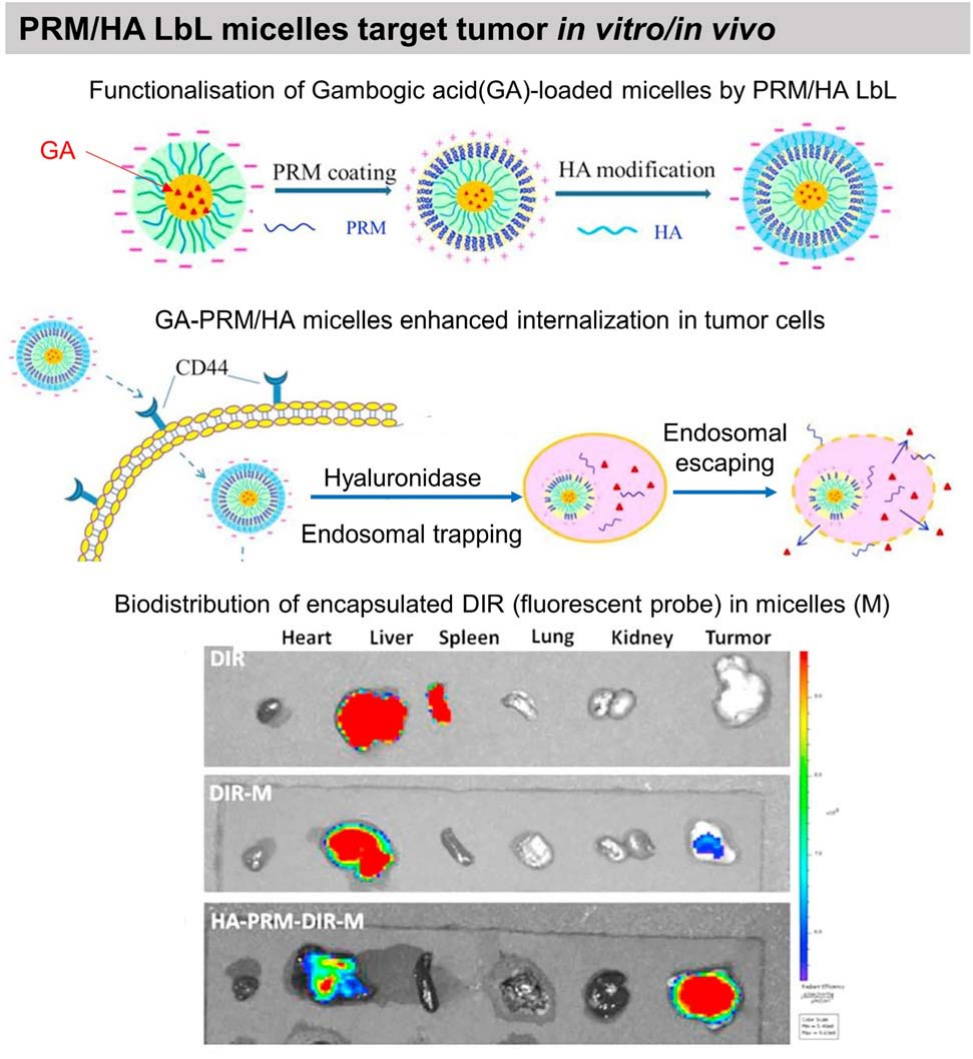
Drug release by enzymatic degradation of LbL films. Scheme of PRM/HA LbL coated bioactive micelles for targeting tumours, and biodistribution *in vivo*. This figure has been adapted from ref. 193 with permission from Elsevier, copyright 2018.

Gambogic acid, a bioactive species, loaded micelles were coated with PRM/HA LbL film. The LbL coating enhanced the internalisation of the micelles by human lung adenocarcinoma, where they undergo the removal of HA in a hyaluronidase (HAase)-rich tumour microenvironment and exposed the PRM that activates the “proton sponge” effect. Thus, showing targeted anticancer activity towards human lung adenocarcinoma (A549) tumour xenografts in nude mice *in vivo* (Fig. 11).^193^ Localized gene delivery from the LbL films was studied through an enzymatic trigger. Single-strand DNA (ssDNA) was delivered from a formaldehyde-induced covalently bonded ssDNA/BSA LbL film *via* proteinase K degradation. The amount of ssDNA and the protein loading was increased by increasing the number of layers.^183^ DNA was delivered from PRM/DNA LbL films under the action of α-chymotrypsin enzymatic degradation.^198^

### Release by dual stimuli: pH and enzymes

In contrast to the desired sustained drug release profiles, in particular cases like cancer treatment and vaccines, a burst release of the drug after reaching the target site might be more efficient. Dual stimuli-responsive (pH and enzyme) PRM/HEP LbL capsules were loaded with Dox at pH 5 for cancer treatment. Once internalized by the MCF-7 breast cancer cells, the capsules disintegrate inside the cells, leading to a burst release of the Dox and subsequent cell death.^194^ PRM-carboxymethylcellulose LbL nanocapsules decorated with Fe_3_O_4_ magnetic nanoparticles were developed to deliver Dox. The presence of an external magnetic field enhanced the capsule uptake at the target site in the Balb/c mouse model *in vivo* with successful Dox delivery under an external magnetic field.^196^

## Bone regeneration

**Scheme 6:**
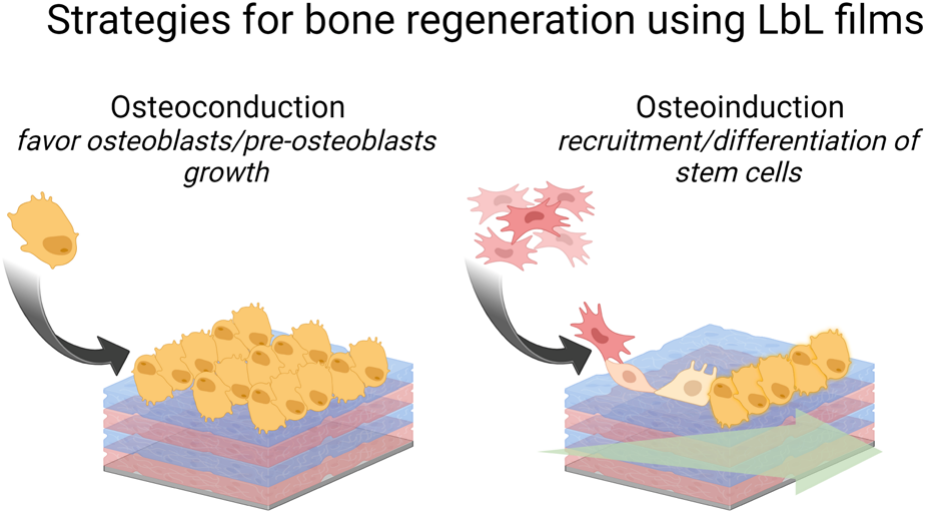
Schematic illustration of LbL strategies to promote bone regeneration.

Failure to osseointegration, the formation of interfacial fibrous tissue between bone and implant, is a major reason for implant aseptic loosening. Several types of implant materials have been functionalized using the LbL technique, such as titanium, polyetheretherketone (PEEK),^204^ poly(l-lactic acid) (PLLA),^205^ stainless steel,^206^ poly(ε-caprolactone) scaffold^207^ and fibres,^208^ or poly(propylene carbonate).^209^ A brief introduction to bone repair requirements including differentiation and the types of cells involved can be found elsewhere.^210^ Subsequently, different LbL films emerge to address various aspects of bone tissue repair, in particular, to regulate cell behaviour on host tissue/implantable devices' interface. Two types of properties have been addressed: osteoconduction which is the ability of osteoblasts or pre-osteoblasts (bone-forming cells) to form new bone over time in the biomaterials or on its surface, and osteoinduction which is the recruitment and differentiation of mesenchymal stem cells (MSCs) and pre-osteoblasts into osteoblastic lineage (Scheme 6). MSCs are multipotent cells that can be differentiated into various cell types, including osteoblasts, chondrocytes, and adipocytes. MSCs differentiated into pre-osteoblasts before their differentiation into osteoblasts.^211^

The differentiation of MSC is commonly obtained thanks to additional growth factors or bioactive calcium phosphates in the cell culture medium or adsorbed in the LbL films.^212^ To this end, cell-adhesive proteins have been incorporated into the LbL films to improve cell adhesion and proliferation by taking advantage of the high adsorbed quantity of proteins, the film dissolution to release bioactive compounds and/or the bioactivity of the partner LbL component to obtain an additional property. During the bone repair process, M1 macrophages secrete mediators that (i) prevent surrounding cells (*i.e.*, osteoblasts and ECs) from growing and proliferating and (ii) activate the osteoclastic bone resorption. Whereas to restrain the fibrous encapsulation of biomaterials, M2 macrophages release mediators that promote the proliferation of adjacent cells involved in osteogenesis, angiogenesis, and osseointegration. Thus, a positive regulation on the M1/M2 phenotype switch appears to be key to the implant osseointegration.

### Osteoconductive LbL films

COL/HA LbL films were used to improve the adhesion of pre-osteoblasts^110^ and osteoblasts on PLLA substrates.^111^ Electrostatically assembled COL/HA LbL films dissolve in physiological conditions. The dissolution of COL/HA LbL films in physiological conditions leads to higher osteogenic protein and gene expression after 14 days, accelerating their differentiation.^213^ The rate of COL/HA LbL film's dissolution in PBS was modulated by a disulfide cross-linking strategy. Incorporation of RGD in cross-linked COL/HA LbL films improved murine pre-osteoblast adhesion with higher bone-specific gene expression levels and matrix mineralization.^214^ The films were obtained using a disulphide RGD peptide subsequently cross-linked by the conversion of free sulfhydryl groups into disulphide linkages. Knowing that COL possesses RGD moieties, the advantage of the presence of RGD on the cross-linker was not discussed in the paper. An *in vivo* comparison between cross-linked and native COL/HA LbL films highlighted the importance of film stability in trabecular bone rabbit models. Cross-linked COL/HA films led to better osseointegration with tight *de novo* bone-implant interfacial contact, reducing the implant loosening, than the native film.^215^ For COL/HA films, the cross-linking strategy resulted in better osteointegration but in the case of COL/Alg the film crosslinking showed terminating layer-dependent property. For example, carbodiimide cross-linked COL/Alg films show significantly higher ALP activity after 12 days of seeding of murine pre-osteoblasts when alginate is the terminating layer. Perhaps, the crosslinking reaction rendered cell-binding domains of COL unavailable for murine pre-osteoblasts.^216^

In comparison to uncoated substrates and Fn monolayer, Fn/PLL LbL films significantly improved human osteoblast-like and pre-osteoblast^134^ adhesion and proliferation.^217,218^ PRM, used for treating heparin overdose or excessive bleeding disorder, gives rise to hydrophilic coatings on silicon substrates with PSS as a polyanionic counterpart. The increase in layer pair number from 20 to 240 directly impacts the surface roughness and Young's modulus in a hydrated state. Such mechanical properties of the coated surfaces appear to significantly influence murine pre-osteoblasts spreading, proliferation, and differentiation with higher ALP activity and mineralization.^219^ Water contact angle (WCA) deals with the wetting behaviour of a surface and indirectly affects mammalian cell adhesion. In the case of superhydrophilicity, *i.e.*, WCA in the range of 0° to 5°, proteins from cell culture medium that can support cell adhesion are thermodynamically unable to adsorb on the surface, giving rise to antifouling surfaces. Similarly, if a surface is hydrophobic, *i.e.*, WCA above 70°, the cell culture medium proteins adsorb on the surface in a denatured manner that also prevents cell adhesion.^220^ Therefore, LbL films are often used to provide well-controlled hydrophilicity to support cell adhesion and proliferation. Thanks to the hydrophilic character of the proteins and polyelectrolytes used, the LbL films decreased the water contact angle of relatively hydrophobic surfaces.

**Fig. 12:**
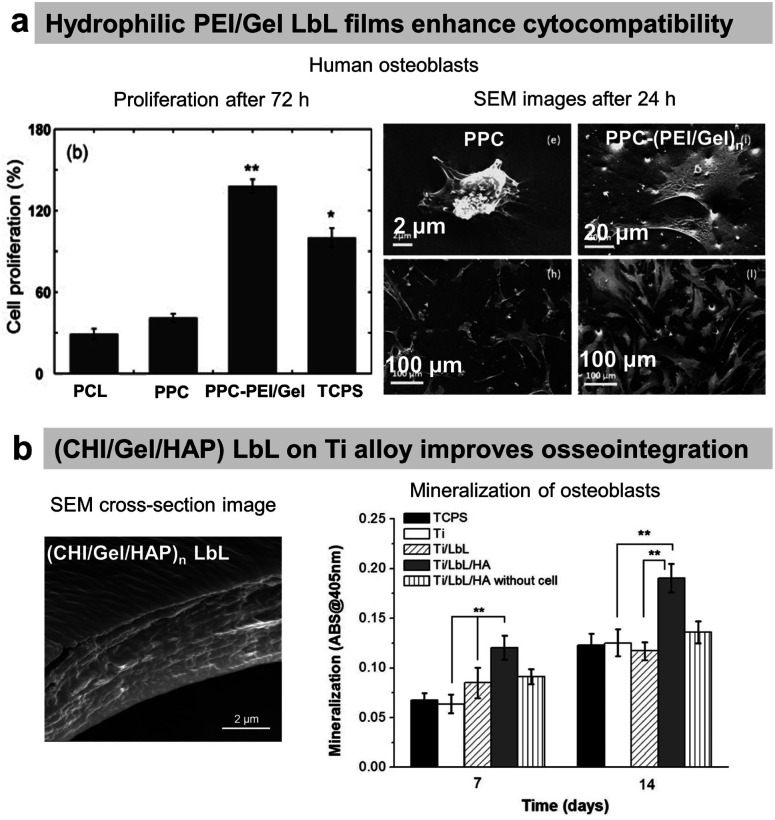
LbL films supporting osteoconduction. (a) Polyethylene imine/gelatin (Gel/PEI) LbL increased the proliferation of human osteoblast cells, thanks to the hydrophilic nature of the LbL film. This figure has been adapted from ref. 209 with permission from Elsevier, copyright 2012. (b) Chitosan/gelatin/hydroxyapatite (CHI/Gel/HAP) composite LbL films on titanium alloy, a cross-section of the films by SEM, and mineralisation of osteoblasts after 7 and 14 days of culture. This figure has been adapted from ref. 221 with permission from Elsevier, copyright 2014.

By increasing the hydrophilicity of poly(propylene carbonate) (PPC), *i.e.*, decreasing the water contact angle from 70° to 58°, Gel/PEI LbL films enhanced the proliferation of both fibroblasts and osteoblasts (Fig. 12a).^209^ Gel/CHI LbL films were extensively used, due to the osteoconductive properties of CHI and the non-immunogenic character of Gel, as platforms for the controlled release of bioactive agents. Similarly, to COL/HA films, Gel/CHI LbL films were built in acidic pH, due to CHI solubility limitation, and became unstable in a physiological medium due to the decrease in electrostatic interactions between partners. After cross-linking *via* carbodiimide chemistry, these films showed better adhesion, proliferation, and viability of osteoblasts in comparison to the uncoated titanium substrate.^222^ Unstable in PBS, Gel/CHI LbL films were exploited for the incorporation and subsequent release of different bioactive agents. The release of Zn ions, below its toxic concentration (2 ppm), improved osteoblasts proliferation and activity up to 7 days, and showed antibacterial activity by reducing *S. aureus* and *E. coli* adhesion.^223^ β-Estradiol, known to upregulate osteoblast maturation and to reduce osteoclast growth, was loaded into mesoporous silica nanoparticles coated by Gel/CHI LbL films and embedded in the same degradable LbL films.^224^ Adhered osteoblasts were able to uptake the drug-loaded nanocarriers, thus displaying higher mineralization than on Gel/CHI films without the loaded nanoparticles. Titanium nanotube usually obtained by electrochemical anodization were reported to significantly improve cell spreading and adhesion.^225^ They can be excellent carriers of bioactive molecules. Icariin, a natural flavonoid from *Epimedium* herbs, potent to treat osteoporosis and inhibit osteoclast differentiation, was delivered from titanium nanotubes coated by the degradable Gel/CHI LbL films. In physiological conditions, the films delayed the release of Icariin up to 5 days leading to higher osteoblast proliferation than the uncoated Icariin loaded titanium nanotube.^226^ Polyaniline-modified CHI (CHI-PANI) was used with Gel in LbL manner to obtain multifunctional antibacterial activity and osteoconductive properties.^227^ Methacrylamide modified Gel/*N*-halanine modified poly(*N*,*N*′-methylene bis(acrylamide)) LbL films were deposited on BMP-2 loaded titanium nanotubes to obtain a pH triggered release of BMP-2 to favour osteoblasts viability as well as antibacterial properties towards *S. aureus* and *E. coli*.^228^ The antibacterial activity is due to *N*-halamines presenting an oxidation state of the chloride ions, targeting thiol groups or amino groups of bacterial proteins, leading to growth inhibition or inactivation. Hydroxyapatite (HAP), a mineral that comprises approximately 70% of the bone mass, is naturally found as nanoneedles. Several layers of HAP nanofibers were sandwiched in Gel/CHI LbL films to obtain a lamellar stack hybrid film (Fig. 12b). These hybrid films improved osteoblast migration and significant mineralization for up to 14 days.^221^

### Osteoinductive LbL films

The choice of LbL partners is crucial for the desired biological response. COL/HEP LbL films coated on PLLA substrates allowed to reduce human MSCs proliferation whilst stimulating their differentiation and mineralization after 28 days which provided osteoinductive environments. Here, HEP interacts with telopeptide regions of COL due to interlayer mobility/diffusion within LbL film and blocks the cell-binding domains of COL that cause lower cell adhesion.^205^ A comparative study in which COL was assembled with native or oxidized HA or CS showed that the molecular composition of LbL films impacted not only the physicochemical properties of the films but also the adhesion of human adipose-derived MSCs on the films.^229^ A favourable microenvironment was obtained with COL/CS films with a higher COL remodelling by the MSCs. This was attributed to a higher amount of COL deposition in COL/CS film. PLA films were functionalized by cross-linked COL/HA embedding silicon-carbonated HAP nanoparticles, leading to better cell attachment, proliferation, and osteogenic activity of human MSCs than on unmodified PLA.^230^

**Fig. 13:**
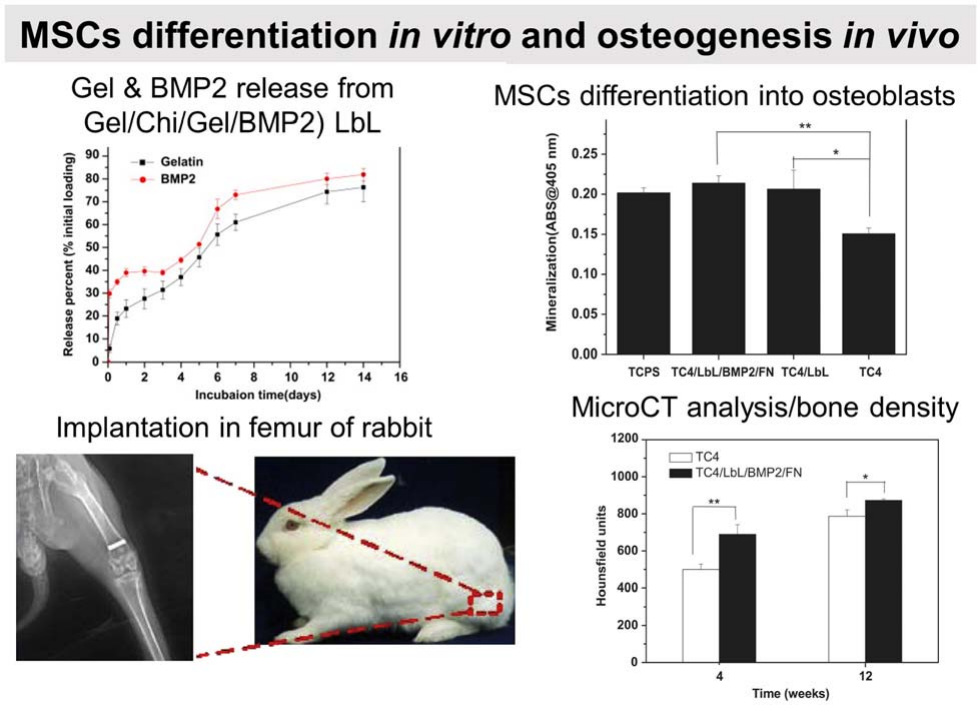
LbL films supporting osteoinduction. Gel/CHI/Gel/BMP2 LbL films were built on a titanium alloy rod for implantation in rabbit femur *in vivo*. Gel and BPM2 were released in a physiological medium. LbL-coated surfaces show better mesenchymal stem cell mineralization, and bone formation (MicroCT and histochemical analysis). This figure has been adapted from ref. 231 with permission from Elsevier, copyright 2012.

Osteoinduction properties can also be obtained by the action of growth factors, proteins that stimulate the growth of specific tissues, on MSCs. Bone Morphogenetic Protein 2 (BMP-2) is a multi-functional growth factor released during bone growth or healing. The embedding of BMP-2 in Gel/CHI LbL allowed better differentiation of MSCs compared to bare alloy substrates (Fig. 13).^231^ Although, the authors did not report any significant difference between Gel/CHI, LbL/BPM-2/Fn, and the TCPS group (see mineralization assay), in the physiological medium, the sustained release of Gel and BPM-2 over 14 days induced higher osteoblastic protein expressions in comparison to the uncoated substrate.^231^ A more pronounced formation of *de novo* bone was observed *in vivo* in the rabbit femur model thanks to BMP-2 release. The great advantage of LbL is the possibility to have multiple properties with the same film. A double functionalization of the porous titanium scaffold was achieved by Gel/CHI LbL films and ended by Gel/BMP-2 layer and CHI/antibiotic layer. This led to higher ALP expression (2-fold), matrix mineralization (4-fold) as well as antibacterial activity against *S. aureus* up to 8 log reduction in planktonic and adherent bacteria in comparison to the uncoated scaffold. *In vivo* subcutaneous implantation in rats revealed the connective tissue formation with no foreign body response after 8 weeks.^232^ Based on the assumption that insulin growth factor (IGF) plays an important role in maintaining bone strength, IGF adsorbed as a last layer on Gel/CHI LbL demonstrated significantly higher osteogenic differentiation of MSCs and new bone formation in rat with osteoporosis.^233^

### Osteo-immunomodulatory LbL films

In this context, Zhao *et al.*, empowered an interconnected nanoporous titanium (Ti) network with polydopamine forming a polydopamine anchoring layer then immobilized COL through three reactions: the Schiff base reaction between the NH_2_ of COL and quinone of polydopamine, the Michael-type addition reaction between the NH_2_ of COL and catechol of polydopamine, and the amide bond (CO–NH) formation reaction between the COOH of COL and NH_2_ of polydopamine. The increase in the COL content was achieved by COL/HA LbL. In contrast to the raw Ti network, which favoured M1 macrophage polarization, the COL/HA coating significantly downregulates the transcription of M1 macrophage genes (*i.e.*, iNOS, CD11C, CD86, and CCR-7) and upregulates the transcription of M2 macrophage genes (*i.e.*, CD163, CD206, IL-10, and arginine-1).^234^

Gel/CHI LbL loaded with SEW2871, a macrophage recruitment agent, was built on dopamine-modified Ti. In addition to the increase in RAW264.7 cells *in vitro* migration, SEW2871 in LbL film promotes the M2 phenotype. *In vivo*, better early recruitment of endogenous macrophages at the implants–bone interface was observed in the implanted SEW2871 Gel/CHI-coated Ti *versus* raw Ti. Suppression of CD86 positive M1 macrophage and a better expression of CD206 positive M2 macrophages after 7 days of implantation were observed close to SEW2871 Gel/CHI coated Ti in comparison with raw Ti.^235^ As promising alternative biomaterials to conventional metals in bone reconstructive surgery, poly(etheretherketone) (PEEK) possesses superior characteristics including excellent mechanical properties, good chemical stability, and biocompatibility, as well as natural radiolucency. However, macrophages persist in the M1 state on the PEEK surface, leading to fusion into multinucleated giant cells, which contribute to the formation of fibrous encapsulation and inferior osseointegration. O_2_ plasma-treated PEEK coated with PAH/poly(acrylic acid) LbL film built at pH 1.8 and ended by PAH inhibits the early M1 polarization of macrophages, promotes the M2 polarization with prolonged culture time, and suppress the osteoclast formation.^204^

## Neural interfaces

**Scheme 7:**
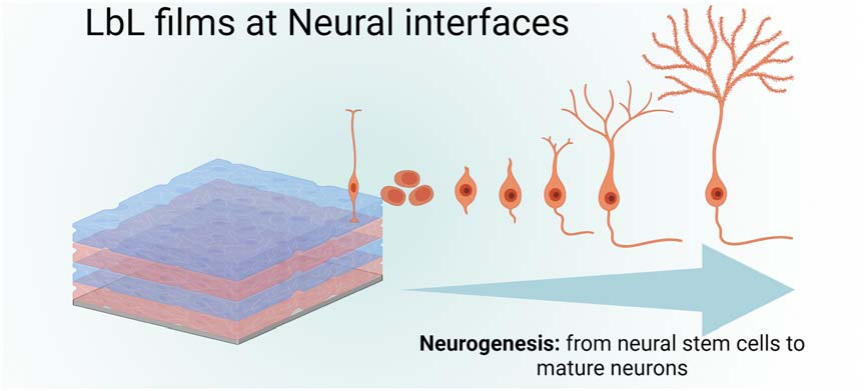
Schematic illustration of neurogenesis, *i.e.*, differentiation cascade of neural stem cells to form mature neurons, onto LbL films.

Protein-based LbL films can provide physicochemical as well as bio-specific cues for attachment, proliferation, and differentiation of neural cells (Scheme 7).

**Fig. 14:**
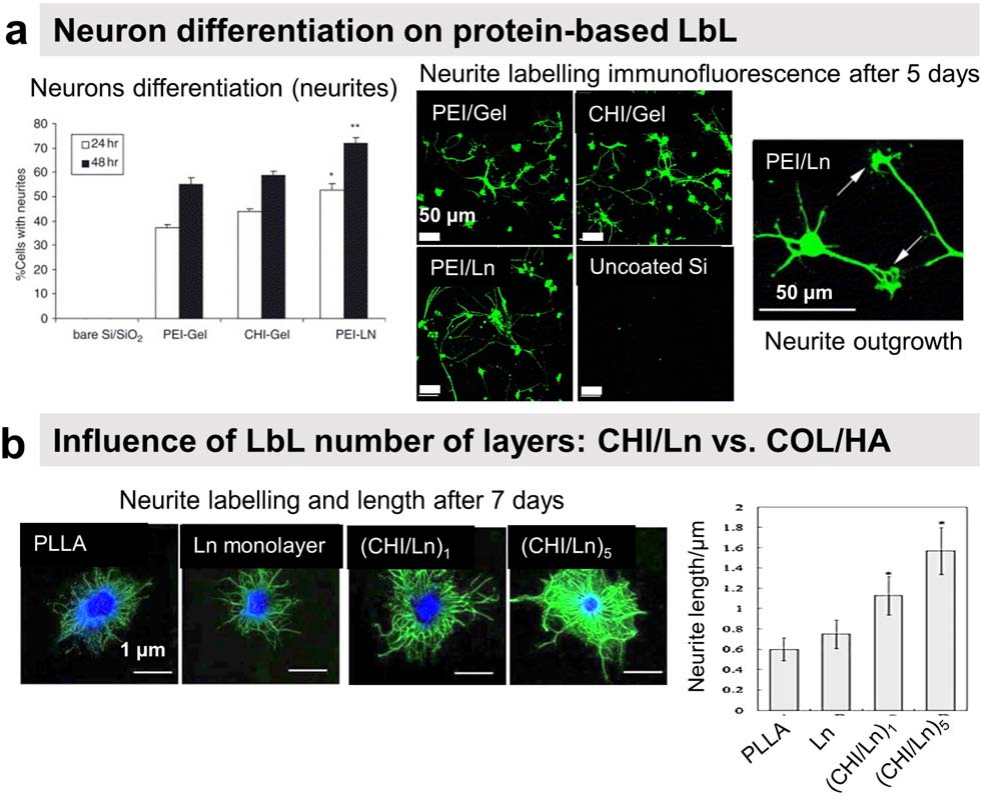
LbL films promote neural cell adhesion and differentiation. (a) Silicon microelectrode arrays (Si MEAs) coated with various LbL films, with compositions such as PEI/Gel, CHI/Gel, and PEI/laminin (Ln), show better chick cortical neuron differentiation only when using protein-based LbL, *i.e.*, Gel or Ln, among which PEI/Ln was the best composition. This figure has been adapted from ref. 237 with permission from Elsevier, copyright 2005. (b) Chitosan/laminin (CHI/Ln) LbL film on poly-l-lactide (PLLA) electrospun scaffold, with no preferential orientation of the nanofibers, increased the neurite outgrowth of neurons after 7 days. Increasing the number of bilayers from 1 to 5 enhanced the neurite outgrowth, thanks to a higher amount of Ln incorporated into the LbL film. This figure has been adapted from ref. 238 with permission from John Wiley and Sons, copyright 2013.

Laminin (Ln), a tri-peptide glycoprotein, is known to favour neurite outgrowth by integrin-specific interactions. Fn/poly(d-lysine) (PDL) and Ln/PDL were built on silicon rubber, functionalized by (PSS/PEI)_3_ precursor layer. Stable in PBS for 30 days, both films showed comparable neuronal cell attachment, differentiation, and neurite outgrowth with an elongated shape.^236^ Silicon microelectrode arrays (Si MEAs) can detect neural activity *in vivo*. However, in terms of impedance, their long-term performance is compromised by fibrous encapsulation and scar tissue development. Si MEAs were coated with Ln/PEI, Gel/PEI, and Gel/CHI LbL films (Fig. 14a). In comparison to Gel/PEI and Gel/CHI LbL films, Ln/PEI LbL films coated on Si MEA showed the highest attached neuron density (4 h) and neurite outgrowth with clearly observable growth cones and network connections. The cell spreading was better and neurites were longer and thicker. Gel/CHI showed the least attached neuron density in comparison to Gel/PEI. The authors suggested that the interpenetration of underlying bulky CHI chains renders fewer Gel sites available for neuron attachment, in comparison to linear PEI chains. Nevertheless, the neurite outgrowth was comparable to Gel/PEI after 24 and 48 h.^237^ Similar results were reported for Gel/CHI LbL films coated on PLLA electrospun fibres. The number and length of dendrites (a differentiated form of neurons responsible for signal transfer) were higher with Gel/CHI multilayers irrespective of the ending layer compared to control monolayers of Gel or CHI. Ln was also assembled with CHI in a LbL manner to coat PLLA electrospun fibres. Ln/CHI coatings were stable in PBS at 37 °C with less than 10% loss of Ln after 20 days. The neuronal cell adhesion, viability, neurite length, and proliferation of dorsal root ganglia neurons improved by increasing the number of Ln/CHI bilayers (Fig. 14b) as well as for neuronal stem cells. The better performance of Ln/CHI films is associated with the higher amounts of laminin incorporated with each bilayer.^238^

In the case of COL/HA LbL, neuronal cell density, and neurite length decreased with the increase of layers due probably to higher film roughness/heterogeneity.^239^ The adhesion and outgrowth of hippocampal and cortical neurons showed a preference towards HA/PAH and COL/HA LbL films, respectively. Cortical neural prostheses are used to record and translate neural signals into movement commands for paralyzed patients. These electrodes often fail to ensure their role due to inflammation and neuronal loss around the implant. To stimulate neurite outgrowth, Gel/neurotrophin nerve growth factor (NGF) based LbL coatings were deposited on the prostheses. In contrast to Hep/NGF and DS/NGF LbL films, a higher loading and sustained delivery of NGF were obtained for up to 2 weeks under PBS degradation triggering upregulating of neurite outgrowth from PC12 cells.^175^

## Current status, challenges, and future research directions

Since 1992, layer-by-layer (LbL) technology has been used to assemble polyelectrolytes, natural polymers, and proteins for various healthcare applications. Unlike traditional polyelectrolyte LbL films, there is a lack of in-depth understanding of the structure and dynamics of protein-based LbL films and their correlation to the intended biological function. Traditional materials characterization techniques based on light, electron, or X-ray are limited by spatial resolution, sensitivity, sample size, and the need for sample preparation. Despite the potential benefits, the use of ECM-derived proteins in LbL assembly is limited by their structural complexity. As natural polymers, proteins can vary in molecular weight, leading to batch-to-batch variability and contamination issues, especially when produced in *E. coli*. When assembled in LbL, proteins can experience denaturation, structural changes, and loss of biological activity due to complexation with the partner material. Optimization of assembly conditions and choice of LbL partner is therefore necessary for controlled adsorption, orientation, and organization of proteins. Attempts have been made to manipulate the complex surface charge properties of proteins, such as the use of protein nanoparticles/crystals and protein-polymer complexes. Engineered proteins with enhanced stability, specificity, and functionality tuneable properties could overcome some of the limitations associated with native proteins, facilitating their integration into LbL films. However, protein production and purification can be expensive, tedious, and time-consuming tasks. In addition to the cost and availability of proteins in large quantities, there are still some limitations to overcome for commercial scale-up applications, such as difficult control of film uniformity (in terms of thickness, roughness, and architecture), extended build-up time (for dip coating), batch-to-batch variability, industrial adaptability of equipment and its automation for film production, and quality control. In addition, the incorporation of proteins into LbL films on a commercial scale may raise regulatory concerns regarding safety, potential contamination, and biocompatibility. Therefore, it is essential to maintain a sterile environment (such as a clean room) or choose the right sterilization methods (heat or irradiation can damage proteins). Finally, for practical applications, a robust and stable LbL film is often required, which is achieved by chemical crosslinking, which can adversely affect the biological function of proteins. In this regard, maintaining the biological activity of proteins in LbL films can be challenging.

## Conclusions

The use of proteins as components of LbL films makes it possible to impart various biological properties to biomaterial surfaces using a simple method, performed at room temperature in water, without chemical modification. The LbL films could be antibacterial, promote or prevent mammalian cell adhesion/differentiation, or deliver drugs/genes. First, all protein-based films can be used to deliver (macro)molecules (antibacterial agent, drug, gene, *etc.*) by enzymatic degradation by α-chemotrypsin or specific enzymes (collagenase). Carrying pro-adhesive moieties (RGD sequence), COL, Gel and Fn-based films promote fibroblast, endothelial, and pre-osteoblast adhesion with an influence of the final layer. COL-based films are also osteoinductive, promoting the recruitment and differentiation of stem cells into the osteoblastic lineage. Ln-based films have been specifically used to promote neuronal outgrowth and maturation through integrin-specific interactions. BSA, a plasma protein with anti-thrombogenic properties, was also used to reduce non-specific platelet adhesion. PRM-based films were mainly used for gene transfection. Antibacterial enzymes were incorporated to achieve antibacterial properties due to their ability to degrade biofilm components (proteoglycans) or quorum-sensing molecules. Specific proteins such as immunoglobulins or growth factors have been used for recognition or stem cell differentiation. Gel-based films have been used to coat drug nanocrystals for sustained release in a physiological medium while avoiding burst release. By a judicious choice of the partners and association, multifunctionality can be obtained. This could be a nice and interesting perspective of such LbL films. Predicting LbL properties, in particular protein-based films, remains a challenge. Machine learning methodologies emerge and potentially revolutionize the field. Recently, Vrana and co-workers used literature data and in-house generated experimental results for coating thickness prediction. These studies could be the first steps for the prediction of biological properties.^240,241^

## Abbreviations

AFM: Atomic force microscopy
Alg: Alginate
BMP2: Bone morphogenetic protein 2
BSA: Bovine serum albumin
COL: Type I collagen
CHI: Chitosan
CS: Chondroitin sulfate
CLSM: Confocal laser scanning microscopy
DAPI: 4,6-Diamidino-2-phenylindole
DNA: Deoxyribonucleic acid
DOP: Dopamine
DOX: Doxorubicin
DS: Dextran sulphate
EC: Endothelial cells
*E. coli*
: *Escherichia coli*
EGCG: Epigallocatechin gallate
ECM: Extracellular matrix
ELR: Elastin-like recombinamers
EPC: Endothelial progenitor cell
Fn: Fibronectin
FDA: U. S. Food and Drug Administration
FGF: Fibroblast growth factor
Gel: Gelatin
GO: Graphene oxide
HA: Hyaluronic acid
HAP: Hydroxyapatite
HEP: Heparin
HEPES: 4-(2-Hydroxyethyl)-1-piperazineethanesulfonic acid
HSA: Human serum albumin
HGFs: Human gingival fibroblasts
HMDS: Hexamethyldisilazane
HUVEC: Human umbilical vein endothelial cells
H_2_O_2_: Hydrogen peroxide
IGF: Insulin growth factor
IgG: Immunoglobulin G
Ly: Lysozyme
Ln: Laminin
LbL: Layer-by-layer
MRSA: Methicillin-resistant *Staphylococcus aureus*
MH: Minocycline hydrochloride
MSCs: Mesenchymal stem cells
NP: Nanoparticle
NaCl: Sodium chloride
NaOH: Sodium hydroxide
NGF: Nerve growth factor
NO: Nitric oxide
PAA: Poly(acrylic acid)
PAAm: Polyacrylamide
PAH: Poly(allylamine hydrochloride)
PBS: Phosphate buffered saline
PDA: Polydopamine
PDADMAC: Poly(diallyl dimethyl ammonium chloride)
PDL: Poly(d-lysine)
pDNA: plasmid DNA
PEEK: polyetheretherketone
PEG: Poly(ethylene glycol)
PEI: Polyethylenimine
PET: Poly(ethylene terephtalate)
PGA: Poly(l-glutamic acid)
PLL: Poly(l-lysine)
PLLA: Poly(l-lactic acid)
PAR: poly(l-arginine)
PLGA: Poly lactic-*co*-glycolic acid
PMMA: Poly(methacrylic acid)
PPC: Poly(propylene carbonate)
PRM: Protamine
PSS: Poly(styrene sulfonate)
PVAm: Poly(vinylamine)
pI: Isoelectric point
*P. gingivalis*
: *Porphyromonas gingivalis*
PTFE: Polytetrafluoroethylene
PVC: Poly(vinyl chloride)
RGD: Arginine–glycine–aspartic acid
QAC: Quaternary ammonium compounds
*S. aureus*
: *Staphylococcus aureus*
SF: Silk fibroin
SEM: Scanning electron microscopy
SiMEA: Silicon microelectrode arrays
SMC: Smooth muscle cells
SS: Stainless steel
TA: Tannic acid
TCPS: Tissue culture poly(styrene)
Ti: Titanium
XPS: X-ray photoelectron spectroscopy

## Author contributions

Muhammad Haseeb Iqbal: writing original draft, Halima Kerdjoudj: writing – review & editing, and Fouzia Boulmedais: supervision, resources, funding acquisition, writing – review & editing, project administration.

## Conflicts of interest

There are no conflicts to declare.
